# Single-cell transcriptome of early embryos and cultured embryonic stem cells of cynomolgus monkeys

**DOI:** 10.1038/sdata.2017.67

**Published:** 2017-06-20

**Authors:** Tomonori Nakamura, Yukihiro Yabuta, Ikuhiro Okamoto, Kotaro Sasaki, Chizuru Iwatani, Hideaki Tsuchiya, Mitinori Saitou

**Affiliations:** 1 Department of Anatomy and Cell Biology, Graduate School of Medicine, Kyoto University, Yoshida-Konoe-cho, Sakyo-ku, Kyoto 606-8501, Japan; 2 JST, ERATO, Yoshida-Konoe-cho, Sakyo-ku, Kyoto 606-8501, Japan; 3 Research Center for Animal Life Science, Shiga University of Medical Science, Seta-Tsukinowa-cho, Otsu, Shiga 520-2192, Japan; 4 Center for iPS Cell Research and Application, Kyoto University, 53 Kawahara-cho, Shogoin, Sakyo-ku, Kyoto 606-8507, Japan; 5 Institute for Integrated Cell-Material Sciences, Kyoto University, Yoshida-Ushinomiya-cho, Sakyo-ku, Kyoto 606-8501, Japan

**Keywords:** RNA sequencing, Pluripotent stem cells, Germline development, Embryology

## Abstract

In mammals, the development of pluripotency and specification of primordial germ cells (PGCs) have been studied predominantly using mice as a model organism. However, divergences among mammalian species for such processes have begun to be recognized. Between humans and mice, pre-implantation development appears relatively similar, but the manner and morphology of post-implantation development are significantly different. Nevertheless, the embryogenesis just after implantation in primates, including the specification of PGCs, has been unexplored due to the difficulties in analyzing the embryos at relevant developmental stages. Here, we present a comprehensive single-cell transcriptome dataset of pre- and early post-implantation embryo cells, PGCs and embryonic stem cells (ESCs) of cynomolgus monkeys as a model of higher primates. The identities of each transcriptome were also validated rigorously by other way such as immunofluorescent analysis. The information reported here will serve as a foundation for our understanding of a wide range of processes in the developmental biology of primates, including humans.

## Background & Summary

For more than half a century, mice have been exploited as a representative model organism for mammalian development and physiology. The results acquired from these researches have greatly contributed to our understanding of such processes as well as diseased states. Nonetheless, it has been widely recognized that there are significant differences in development and physiology between mice and humans.

The epiblast (EPI) is made up of cells specified during the peri-implantation period of early embryogenesis and differentiates into three primary germ layers and the germ cell lineages; the EPI therefore bears the pluripotency. Even within such a transient period, the EPI cells show dynamic changes in pluripotency from a so-called naive to a primed state^1^, and both states in mice have been captured *in vitro*: the naïve state is replicated in ESCs/induced pluripotent stem cells (iPSCs), which have essentially the same status as the pre-implantation EPI *in vivo*^2^, while the primed state is replicated in epiblast stem cells (EpiSCs), which are derived from the post-implantation EPI and are homologs of the EPI of the gastrula^3,4,5^. On the other hand, although human ESCs/iPSCs have been established, their characteristics—including morphology, culture requirements, and the molecular networks underlying the pluripotent state—have been considered to more closely resemble those of mouse EpiSCs. The underlying mechanisms of these differences have remained unresolved, in part due to the lack of *in vivo* analyses of human/primate early post-implantation development.

The PGCs emerge from the posterior EPI at the onset of gastrulation in mice^6^. The specification process of mouse PGCs has been extensively studied and was reconstructed *in vitro*^7^. Remarkably, the resultant cells (PGC-like cells: PGCLCs) have the ability to produce functional gametes, and the dynamics of PGCLC specification is highly similar to that *in vivo*. In humans, PGCLCs have also been induced from human ESCs/iPSCs^8,9^, but it has still remained unclear whether their properties recapitulate those *in vivo*, again due to the lack of *in vivo* information.

Here, we present a comprehensive transcriptome dataset at the single-cell level from pre- and post-implantation embryo cells, PGCs and ESCs of cynomolgus monkeys, one of the primates amenable to experiment and most closely related to humans. We employed the single-cell mRNA 3′ end sequencing (SC3-seq), which was designed to enrich the reads of the 3′ end of transcripts and enables highly quantitative and effective analysis^10^. We successfully amplified a total of 1,241 single-cell cDNAs and generated 474 transcriptomes (Table 1 (available online only)). The qualities of the transcriptomes and the representations of gene expression profile were validated by qPCR. The sample annotations were defined comprehensively by comparing the expression of key genes in transcriptome data with that obtained through the histological analysis such as immunofluorescent analysis and/or in situ hybridization^11,12^. Thus, the dataset in this Data Descriptor defined the first comprehensive molecular dynamics of primate early development, including early post-implantation embryogenesis, and will provide a foundation for future studies of primate development.

## Methods

The description of the method is extended from the related research manuscripts^11,12^.

### Experimental study design

**Figure 1:**
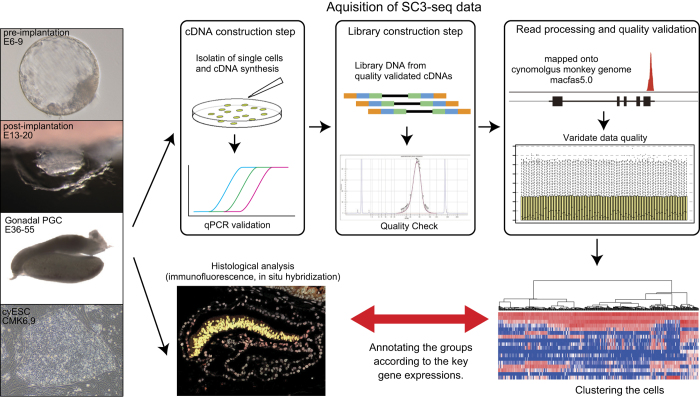
Overview of the study design and work flow. The overall experimental design and work flow. The isolated cells dissociated from each samples were picked up for the cDNA synthesis. Then the quality and key gene expression of the generated cDNAs were examined by qPCR and the selected samples were used for the library construction. The libraries were sequenced and the resultant reads were mapped onto the cynomolgus monkey genome. The global distribution of gene expression in each cell was validated. Finally, the annotations of cell clusters were defined by comparing the gene expression patterns of the transcriptome and histological analysis such as immunofluorescent analysis.

The overall experimental design is illustrated in Fig. 1. For the generation of SC3-seq data, single cells were prepared from pre- and post-implantation embryos, genital ridges and ESCs. They were picked manually and the SC3-seq cDNAs were amplified. After the quality validation and the selection of cDNAs, the DNA libraries for massive parallel sequencers were constructed (see the results of prior qPCR analyses^11,12^). The sequence reads were acquired by SOLiD5500xl and were mapped onto the cynomolgus monkey genome, Macaca fascicularis 5.0 (MacFas5.0). Then, the reads count was converted into reads per million (RPM). Finally, we verified the global distributions of gene expression and obtained quality-validated transcriptomes. Generally, single-cell transcriptome analysis drops off the positional information. Therefore, the expression patterns of key genes were also examined by immunofluorescent analysis or in situ hybridization of their expression, and each transcriptome data point was annotated accurately by comparison with its histologically examined counterpart^11,12^.

### Animals

The experimental procedures were approved by the Animal Care and Use Committee of Shiga University of Medical Science, and the methods were carried out in accordance with the approved guidelines. The procedures in cynomolgus monkeys for housing, oocyte collection, intra- cytoplasmic sperm injection (ICSI), pre-implantation embryo culture, and transfer of pre- implantation embryos into foster mothers were performed as described previously with some modifications^9, 11,12,13,14^. Briefly, monkeys were housed individually in appropriate cages, and the light cycle consisted of 12 h of artificial light from 8 AM to 8 PM. Temperature and humidity in the animal rooms were maintained at 25±2 °C and 50±5%, respectively. Each animal was fed 20 g kg^‒1^ of body weight of commercial pellet monkey chow (CMK-1; CLEA Japan Inc., Tokyo, Japan) in the morning, supplemented with 20–50 g of sweet potato in the afternoon. Water was available *ad libitum*.

For super-ovulation, ovarian stimulation with follicle-stimulating hormone (Gonapure; ASKA) was performed by embedding an implantable and programmable micro-fusion device (iPRECIO; Primetech Corporation) subcutaneously. The day when the ICSI was performed was designated as embryonic day 0 (E0). For embryo transfer, 4 to 5 two-cell to blastocyst-stage embryos were selected and transferred into an appropriate recipient female. For the detection of pregnancy of post-implantation embryos, implanted embryos were monitored by ultrasound scanning. For the collection of early post-implantation embryos (until around E20), the implanted uterus was surgically removed and bisected for the isolation of embryos. For the germ cell collection in gonads, the embryos were delivered by Caesarean section and bisected for the isolation of gonads. All the embryos used in this Data Descriptor are listed in Table 2 (available online only).

### Cell culture

The cynomolgus ESCs [CMK6 (male) and CMK9 (female)] were gifts from H. Suemori^15^. For cultivation on feeders, they were cultured with conventional human ESC medium [DMEM/F12 (D6421; Sigma-Aldrich) supplemented with 20% (vol/vol) of KSR (10828-028; Thermo Fisher Scientific), 1 mM of sodium pyruvate (11360-070; Thermo Fisher Scientific), 2 mM of GlutaMax (35050-061; Thermo Fisher Scientific), 0.1 mM of non-essential amino acids (11143-050; Thermo Fisher Scientific), 0.1 mM of 2-mercaptoethanol (M3148; Sigma-Aldrich), 1,000 U ml^‒1^ of ESGRO mouse LIF (ESG1107; Millipore), and 4 ng ml^‒1^ of recombinant human bFGF (060-04543; Wako Pure Chemical Industries)] on mouse embryonic feeders (MEFs)^16^. For feeder-free cultivation, cynomolgus ESCs were cultured under the same condition as human iPSCs (StemFit; Ajinomoto) on recombinant LAMININ511 (iMatrix511, Nippi), as described previously^17^. All of the cell lines were tested for mycoplasma contamination by MycoAlert (LT07-118; Lonza Japan), according to the manufacturer’s instructions.

### Single-cell preparation

For pre-implantation embryos, the zona pellucida was removed by acid tyrode solution treatment (T1788; Sigma-Aldrich). Then the whole embryo was incubated with 0.25% trypsin/PBS (T4799; Sigma-Aldrich) for around 10 min at 37 °C, then dissociated into single cells by repeated pipetting, and dispersed in 0.1 mg ml^‒1^ of PVA/PBS (P8136; Sigma-Aldrich) for preparation of single-cell cDNAs.

For early post-implantation embryos, the implantation site was dissected out from the uterus and the embryonic fragment containing the EPI, amnion, hypoblast, and yolk-sac endoderm was isolated manually. For isolation of PGCs, in several instances relatively posterior parts of the embryonic fragments were dissected. The embryo information and dissected positions are summarized in Table 1 (available online only). For the embryonic gonads, the genital ridges were dissected out from embryos manually. Then each fragment was incubated with 0.25% trypsin/PBS for around 10 min at 37 °C, dissociated into single cells by repeated pipetting, and dispersed in 0.1 mg ml^‒1^ of PVA/PBS.

For cynomolgus ESCs, the cells were first detached as clumps with CTK solution [0.25% trypsin (15090-046; Thermo Fisher Scientific), 0.1 mg ml^‒1^ of collagenase IV (17104-019; Thermo Fisher Scientific), and 1 mM of CaCl2 (06729-55; Nacalai Tesque)], incubated in TrypLE Select (12563029; Thermo Fisher Scientific) for around 10 min at 37 °C, and dispersed into single cells in 1% (vol/vol) KSR/PBS containing 10 μM of the ROCK inhibitor Y-27632 (257-00511; Wako Pure Chemical Industries)^16^. Cells under the feeder-free condition were directly incubated in TrypLE Select for around 5 min at 37 °C, and dispersed into single cells in 1% (vol/vol) KSR/PBS containing 10 μM of the ROCK inhibitor Y-27632.

### Acquisition of SC3-seq data

The SC3-seq process consists of two steps: the synthesis and amplification of cDNAs from isolated single cells, and the construction of a DNA library for sequencing by the SOLiD5500xl sequencer. The first step, the synthesis and amplification of the single-cell cDNA, was performed essentially as described previously^10^. Briefly, cells were picked up manually into the lysis buffer containing tagged dT primer [V1dT(24)], and lysed by heating. Then the Reverse transcription buffer was added and the 800~1,500 nt cDNA strand was synthesized with short reaction time (5 min). The reaction mixture contained an excess amount of tagged dT primers, which would have interrupted proper amplification. Therefore, the remaining dT primers were digested by exonuclease. Next, in order to add the poly A tail at the end of the synthesized cDNA strand, a terminal deoxynucleotidyl transferase mixture was added into the reaction. Then the second cDNA strand with another tag was synthesized by using a V3-tagged dT primer [V3(dT)24]. Finally, the synthesized double-strand cDNAs were amplified by PCR using V1 and V3 sequences, and the cDNAs with V3 and V1 tags at the 5′ and 3′ sides of the mRNA were obtained.

Before the construction of the DNA library, the quality of the amplified cDNAs was evaluated by examining the Ct values of the qPCR of several endogenous genes and by examining the distribution of the lengths of cDNA fragments using a LabChip GX (CLS760672; Perkin Elmer) or Bioanalyzer 2,100 (5,067-4,626; Agilent Technologies) system. qPCR was performed using Power SYBR Green PCR Master Mix (4367659; Thermo Fisher Scientific) with a CFX384 real-time qPCR system (Bio-Rad, Hercules, CA) according to the manufacturer’s instructions. According to the pilot experiments, the samples whose Ct values of *GAPDH* were more than 21 tended to have poor quality (data not shown). Therefore, we considered the samples whose Ct values of *GAPDH* and *PPIA* were less than 19 and 20 as those with good quality. Then we chose appropriate samples from good quality cDNAs for library construction based on the combinations of the lineage-specific gene expression (Table 3 (available online only)). For the cells from pre-implantation embryos, NANOG, GATA4 and GATA6 were used for the marker of EPI, hypoblast and hypoblast/Trophectoderm. For the cells from post-implantation embryos, EPI cells were defined as POU5F1(+)/ NANOG(+)/ SOX2(+)/ PRDM14(+)/ T(−)/ GATA4(−), and gastrulating cells were POU5F1(+)/ NANOG(low)/ PRDM14(low)/ some of T, GATA4, GATA6(+). The extraembryonic cells such as visceral endoderm, yolk sac endoderm and extraembryonic mesenchyme were classified as POU5F1(low) and other lineage-specific genes (+). The early PGCs were identified as PRDM1(+)/ TFAP2C(+)/ SOX17(+)/SOX2(−). The late PGCs from embryonic gonads were identified as POU5F1(+)/ NANOG(+)/ TFAP2C(+)/ SOX2(−). Most of the primer sets were designed using Primer-Blast (NCBI) within a distance of 500 base pairs (bp) from the transcription termination sites (TTSs). The primer sets and oligo DNA sequences used in this Data Descriptor are given in Table 3 (available online only).

SC3-seq libraries of quality-checked cDNAs were constructed as described previously^10^. Briefly, the cDNAs were digested into 150–250 bp fragment by sonication (M210, Covaris). Then the damaged cDNA fragments were end-polished by T4 DNA polymerase (M203; NEB) and T4 polynucleotide kinase (M201; NEB). The cDNAs are now expected to be broken into three types of fragments; the ones with the V3-tag (5′ side of the original mRNA), the ones with the V1-tag (3′ side of the original mRNA), and the ones without any tag (internal part of the original mRNA). This fragment mixture was subjected to a one-cycle DNA polymerization step to allocate the internal adaptor sequence (the essential tag for the SOLiD sequencer) at the end of V1 tag. Then the P1-adaptor (another essential tag for the SOLiD sequencer) was added by ligation at the internal adaptor-free end. Finally, the index sequence and P2 tag (tag for the SOLiD sequencer) were added by PCR, and the library DNAs were obtained.

The quality and quantity of the constructed libraries were evaluated by using a LabChip GX or Bioanalyzer 2,100 system, a Qubit dsDNA HS assay kit (Q32851; Thermo Fisher Scientific), and a SOLiD Library TaqMan Quantitation kit (4449639; Thermo Fisher Scientific). The clonal amplification of the libraries on beads by emulsion PCR was performed using a SOLiD EZ Bead System (4449639; Thermo Fisher Scientific) at the E120 scale according to the manufacturer's instruction. The resulting bead libraries were loaded onto flowchips and sequenced for 50 and 5 bp barcode plus Exact Call Chemistry (ECC) on a SOLiD 5500XL system (4449639; Thermo Fisher Scientific).

Note that the SC3-seq method has now been modified to be applicable to the illumina sequencers. The detailed protocol has been recently published^18^.

### Reference sequences

The genome sequence for MacFas5.0 (mfa_ref_Macaca_fascicularis_5.0_X.fa (X: chr1 - chr20, chrX)) and transcript definition file (GFF3) for MacFas5.0 (ref_Macaca_fascicularis_5.0_top_level.gff3) were obtained from the NCBI ftp site. ERCC spike-in sequences were obtained from the Thermo Fisher Scientific website (https://tools.thermofisher.com/content/sfs/manuals/cms_095047.txt).

### Modification of transcript definition file

The SC3-seq protocol enriches ~300 bp from the 3′-end of transcription termination sites (TTSs). Therefore, an inaccurate definition of a TTS, especially one that defines TTS as too far short of the appropriate site, could result in an absence of expression values, even if there are reads accumulated further downstream. To ensure that all possible transcript signals are covered, we extended all 3′-end of TTSs by up to 10 kb in the transcript definition file (GFF3 file, all ‘gene’, ‘transcript’ and ‘exon’ type category, Supplementary File 1) by applying the following rules according to the previous report^10^:

1. Identify genes sharing identical TTSs. Remove all these genes but one at each TTS position.
2. Extend TTS by 10 kb if no gene is found within the targeted area.
3. If the extended TTS hits a transcription start site (TSS) of a downstream gene (same strand), stop the extension at 1 bp upstream of the TSS.
4. If the extended TTS hits a TTS of a downstream gene (opposite strand), stop the extension at the mid of two TTS.
5. Remove all pseudogenes (marked as ‘pseudo=True’), tRNAs or ncRNAs.

Since the gene names in these MacFas5.0 annotations were not fully annotated like their human counterparts, we searched for genomic coordinates of the MacFas5.0 genes on the human genome, hg19, using the LiftOver tool of UCSC, and matched the MacFas5.0 gene name with the human one if the names were different^12^.

### Read processing, mapping and conversion to expression values

All reads were pre-processed by cutadapt v1.11 with ‘-c -e 0.1 -q 20 -n 2 -O 1 -m 30’ options and ‘-a’ and ‘-g’ options for sequence CTCGAGGGCGCGCCGGATCCATATACGCCTTGGCCGTACAGCAG, and -a option for sequence A(20) to remove adaptor and poly-A sequences, and low quality bases^19^. Untrimmed and trimmed reads of 30 bp or longer were mapped onto the MacFas5.0 genome and ERCC spike-in RNA sequences with tophat v2.0.11/bowtie1.0.1 with ‘-bowtie1 -C -no-coverage search’’ options^20^. Mapped reads on the genome and ERCC in the bam file were separated and reads on the genome were processed with cufflinks v2.2.0 with ‘—compatible-hit-norm —no-length-correction—library-type fr-secondstrand’ options and MafFas5.0 reference gene annotation with extended TTSs^21^.

## Data Records

The raw csfasta and QV.qual files were deposited in the Gene Expression Omnibus (GEO) database under acquisition numbers GSE67259, GSE76267 and GSE74767 (Data Citation 1, Data Citation 2 and Data Citation 3). The deposited data also contain the abundance of processed expression data, including the Entrez gene IDs and gene names. The sample information is summarized in Table 1 (available online only).

## Technical Validation

### Verification of the qualities of SC3-seq data

**Figure 2:**
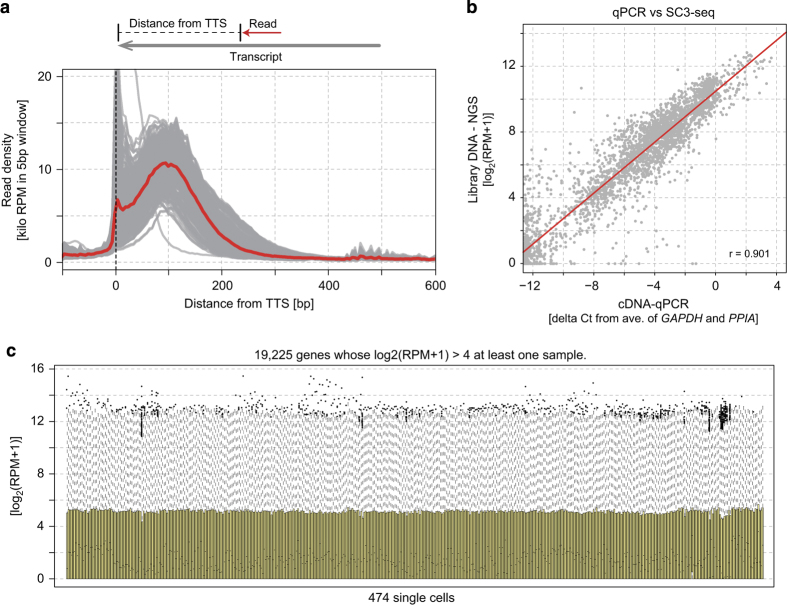
Verification of the qualities of SC3-seq data. (**a**) The SC3-seq track [read density (kilo RPM) plotted against the read position from the annotated TTSs] of all cells (gray) and the average of all cells (red) as indicated above. (**b**) Scatter plot comparison of the gene expressions acquired by qPCR [delta Ct] and by NGS analysis [log_2_(RPM+1)]. The delta Ct values of qPCR analysis were calculated from mean values of *GAPDH* and *PPIA*. The regression line is shown in red, and the correlation coefficient (r) is indicated at the bottom right of the plot. (**c**) Distribution of gene expression levels of all expressed genes. All the expressed genes (19,225 genes) were defined as the genes whose log_2_(RPM+1) values were more than 4 in at least one sample among all 474 cells. The bars at the top, middle and bottom of the box indicate the 75th, 50th, and 25th percentile expression levels, and the top bar of the whisker indicates the expression levels encompassing the expression of 2 s.d. from the median of the genes, respectively.

A total of 474 cDNAs from each single cells were sequenced to a depth of between 0.64–4.73 mega mapped reads (Table 1 (available online only)). According to the previous study^10^, 0.5 mega reads were sufficient for an accurate analysis by SC3-seq. The positions of mapped reads were significantly enriched at the very 3′ ends of mapped genes (Fig. 2a). The comparison of data between cDNAs and subsequent library DNAs indicated that the expression profiles were highly conserved during the experiments (Fig. 2b). Finally, in order to validate the quality of the SC3-seq libraries, we evaluated the profile of expression levels of all the expressed genes in at least one cell (Fig. 2c). Although the single-cell transcriptome is affected by both technical and biological variation, the expression values of the 75th percentile gene showed uniform distribution, indicating that all samples were successfully generated with appropriate qualities.

### Clustering and annotating of the cells

**Figure 3:**
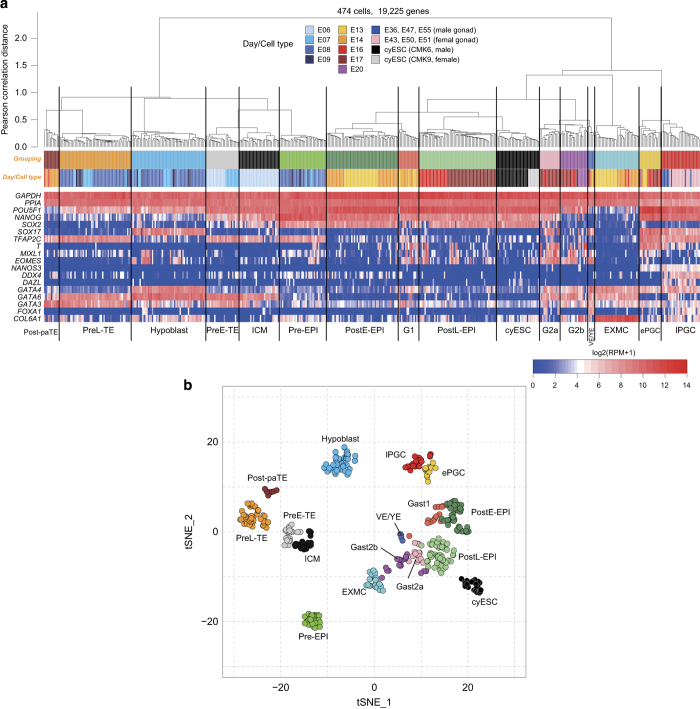
Clustering and annotation of the cells. (**a**) Unsupervised hierarchical clustering (UHC) with all the expressed genes and a heat map of the levels of selected marker genes. The colored bars under the dendrogram indicate grouping (top) and embryonic days/cell type (bottom), respectively. Post-paTE, post-implantation parietal trophectoderm; PreL-TE, pre-implantation late TE; PreE-TE, pre-implantation early TE; ICM, inner cell mass; Pre-EPI, pre-implantation epiblast; PostE-EPI, post-implantation early epiblast; G1, gastrulating cells group 1; PostL-EPI, post-implantation late epiblast; G2a/G2b, gastrulating cells group 1/2; VE/YE, visceral endoderm/yolk sac endoderm; EXMC, extraembryonic mesenchyme; ePGC, early PGC; lPGC, late PGC. (**b**) Plot of two-dimensional t-SNE analysis with all the expressed genes. The color codes is as indicated.

The cells were grouped by unsupervised hierarchical clustering (UHC) using the hclust function of R software with Pearson correlation distance and Ward’s method (ward.D2) (Fig. 3a), and t-SNE analysis using the Rtsne function in the Rtsne package with default parameters^22^ (Fig. 3b). Their annotations were defined by comparing the expression of key genes in transcriptome data with that obtained through the histological analysis^11,12^ and are summarized in Table 1 (available online only).

In UHC analysis, all cells were classified into two large clusters: 6 clusters mainly from pre-implantation embryos and 10 clusters from post-implantation embryos, genital ridges and ESCs (Fig. 3a). In the left side of the dendrogrham in Fig. 3a, the green, blue and orange groups of the pre-implantation cluster consisted of cells from E7 to E9, and each of them expressed the key genes for the pre-implantation epiblast (Pre-EPI) (*NANOG* and *SOX2*), hypoblast (*SOX17, GATA4* and *GATA6*) and late trophectoderm (PreL-TE) (*TFAP2C, GATA3* and *GATA6*) according to the histological analysis^12^. On the other hand, the light gray and dark gray groups in the pre-implantation cluster consisted of the cells from E6 and E7 only, and while the light gray group expressed a high level of trophectoderm-related genes, the dark gray group did not. Consistent with this, in the t-SNE analysis, the cells in the light gray cluster were plotted close to the PreL-TE, while those in the dark gray cluster were located on the side of Pre-EPI and Hypoblast (Fig. 3b). Therefore we annotated them as pre-implantation early trophectoderm (PreE-TE) and inner cell mass (ICM) cells. The brown group, which was derived from post-implantation embryos but located close to PreL-TE in the pre-implantation cluster (Fig. 3a,b), had a similar gene expression pattern to PreL-TE (Fig. 3a), suggesting that the members of the brown group are the derivative of the PreL-TE. Therefore we annotated these cells as post-implantation parietal trophectoderm (Post-paTE) cells.

In the other large cluster in UHC dendrogram (Fig. 3a), the PGCs from genital ridges (late PGCs; lPGCs) were grouped into one cluster (red), and the cells in yellow clusters from the early post-implantation embryo (E13–20) were annotated as early PGCs (ePGCs) because they were clustered with lPGCs both in the UHC dendrogram and t-SNE plot, and also shared characteristic gene expression patterns with lPGCs(Fig. 3a,b). Next, the other clusters of post-implantation cells were classified into two groups according to their *POU5F1* expression (Fig. 3a); *POU5F1* is expressed in embryonic cells during the early post-implantation embryo stage^12^. The light and dark blue groups were annotated as extraembryonic mesenchyme (EXMC) and visceral endoderm/yolk sac endoderm (VE/YE) cells because they showed high and uniform expression of *COL6A1* and *FOXA1* (Fig. 3a), whose expression patterns were confirmed by histological analysis^12^. The remaining clusters were classified as EPI [post-implantation early epiblast, PostE-EPI (E13, 14); post-implantation late epiblast, PostL-EPI (E16,17)] or gastrulating cells (Gast1, 2a, 2b) due to the expression of the pluripotency-associated genes and differentiation-related genes. We could not provide a detailed explanation of the cell types of gastrulating cells due to the highly variable expression of the differentiation-related genes. Consistent with this, the cells in Gast2a and Gast2b were not separated clearly in the t-SNE analysis (Fig. 3b). Both the male and female ESCs (CMK6 and CMK9) were clustered close to PostL-EPI (Fig. 3a,b).

## Additional Information

Tables 1, 2 and 3 are only available in the online version of this paper.

**How to cite this article:** Nakamura, T. *et al.* Single-cell transcriptome of early embryos and cultured embryonic stem cells of cynomolgus monkeys. *Sci. Data* 4:170067 doi: 10.1038/sdata.2017.67 (2017).

**Publisher’s note:** Springer Nature remains neutral with regard to jurisdictional claims in published maps and institutional affiliations.

## Supplementary Material



## Figures and Tables

**Table 1:** Metadata and mapping statistics of SC3-seq analysis

**GSM ID**	**GSM name**	**Cluster in Fgi3**	**order in cluster Fig3**	**Embryonic Day/ passage number**	**cells picked from**	**Embryo name**	**sex**	**GSE ID**	**Total reads**	**SC3-seq mapped reads**	**Mapped_others**	**Control_RNA**	**low_quality_adaptor_polyA**	**Unmapped**
GSM1932351	E16_MS09T43	Post_paTE	1	E16	around embryonic disk	140401ICSIday16	male	GSE74767	6,128,858	**2,774,597**	1,297,685	30,256	1,289,211	737,109
GSM1932349	E14_MS13T86	Post_paTE	2	E14	around embryonic disk	141014ICSIday14A	male	GSE74767	6,429,515	**1,271,723**	351,757	39,719	4,249,933	516,383
GSM1932347	E14_MS09T11	Post_paTE	3	E14	around embryonic disk	140401ICSIday14	male	GSE74767	4,456,818	**1,061,281**	1,264,024	39,322	1,401,801	690,390
GSM1932350	E16_MS09T42	Post_paTE	4	E16	around embryonic disk	140401ICSIday16	male	GSE74767	6,187,993	**1,708,675**	1,319,009	28,864	2,382,646	748,799
GSM1932345	E14_MS09T07	Post_paTE	5	E14	around embryonic disk	140401ICSIday14	male	GSE74767	5,692,791	**2,230,595**	1,137,592	28,407	1,594,158	702,039
GSM1932348	E14_MS13T81	Post_paTE	6	E14	around embryonic disk	141014ICSIday14A	male	GSE74767	5,371,581	**2,182,547**	852,205	15,379	1,665,453	655,997
GSM1932343	E14_MS09T04	Post_paTE	7	E14	around embryonic disk	140401ICSIday14	male	GSE74767	5,434,597	**2,156,596**	1,112,574	23,223	1,468,406	673,798
GSM1932344	E14_MS09T06	Post_paTE	8	E14	around embryonic disk	140401ICSIday14	male	GSE74767	4,864,284	**1,551,471**	902,202	20,353	1,776,915	613,343
GSM1932346	E14_MS09T10	Post_paTE	9	E14	around embryonic disk	140401ICSIday14	male	GSE74767	5,696,608	**2,089,772**	918,302	19,568	2,000,647	668,319
GSM1932341	E14_MS09T01	Post_paTE	10	E14	around embryonic disk	140401ICSIday14	male	GSE74767	6,214,950	**1,955,043**	1,536,175	45,151	1,762,696	915,885
GSM1932342	E14_MS09T02	Post_paTE	11	E14	around embryonic disk	140401ICSIday14	male	GSE74767	5,879,574	**2,345,386**	1,338,822	18,193	1,333,109	844,064
GSM1932533	E08_MS02T34	PreL_TE	12	E08	whole embryo	121107ICSI13_day8	male	GSE74767	6,049,861	**2,871,747**	1,875,055	2,272	729,346	571,441
GSM1932532	E08_MS02T33	PreL_TE	13	E08	whole embryo	121107ICSI13_day8	male	GSE74767	5,060,125	**2,390,667**	1,419,805	1,296	752,289	496,068
GSM1932527	E08_MS02T11	PreL_TE	14	E08	whole embryo	121107ICSI13_day8	male	GSE74767	8,423,512	**4,499,577**	1,807,662	1,951	1,334,781	779,541
GSM1932524	E08_MS02T04	PreL_TE	15	E08	whole embryo	121107ICSI13_day8	male	GSE74767	5,224,475	**2,745,820**	1,234,798	1,390	766,219	476,248
GSM1932528	E08_MS02T19	PreL_TE	16	E08	whole embryo	121107ICSI13_day8	male	GSE74767	5,595,531	**2,740,479**	1,476,266	1,284	834,387	543,115
GSM1932531	E08_MS02T27	PreL_TE	17	E08	whole embryo	121107ICSI13_day8	male	GSE74767	5,887,381	**3,081,912**	1,317,162	1,181	919,368	567,758
GSM1932529	E08_MS02T24	PreL_TE	18	E08	whole embryo	121107ICSI13_day8	male	GSE74767	6,071,248	**3,053,414**	1,341,552	1,242	1,064,737	610,303
GSM1932530	E08_MS02T25	PreL_TE	19	E08	whole embryo	121107ICSI13_day8	male	GSE74767	5,670,112	**2,850,725**	1,377,720	973	888,091	552,603
GSM1932525	E08_MS02T07	PreL_TE	20	E08	whole embryo	121107ICSI13_day8	male	GSE74767	5,778,802	**2,758,330**	1,556,026	1,821	888,837	573,788
GSM1932526	E08_MS02T10	PreL_TE	21	E08	whole embryo	121107ICSI13_day8	male	GSE74767	7,316,886	**3,642,213**	1,799,007	2,012	1,150,717	722,937
GSM1932514	E07_MS03T55	PreL_TE	22	E07	whole embryo	121205ICSI#1day7	male	GSE74767	3,725,478	**1,864,037**	764,322	696	753,139	343,284
GSM1932518	E07_MS03T63	PreL_TE	23	E07	whole embryo	121205ICSI#1day7	male	GSE74767	6,513,999	**2,476,661**	1,071,889	1,384	2,302,184	661,881
GSM1932560	E09_MS03T77	PreL_TE	24	E09	whole embryo	120725ICSIday9	male	GSE74767	4,140,253	**1,685,077**	1,115,324	1,424	790,681	547,747
GSM1932558	E09_MS03T75	PreL_TE	25	E09	whole embryo	120725ICSIday9	male	GSE74767	4,557,502	**2,211,118**	727,286	1,337	1,111,516	506,245
GSM1932559	E09_MS03T76	PreL_TE	26	E09	whole embryo	120725ICSIday9	male	GSE74767	3,390,166	**1,764,821**	660,928	952	593,318	370,147
GSM1932562	E09_MS03T86	PreL_TE	27	E09	whole embryo	120725ICSIday9	male	GSE74767	5,438,653	**2,421,176**	1,136,347	620	1,225,112	655,398
GSM1932561	E09_MS03T84	PreL_TE	28	E09	whole embryo	120725ICSIday9	male	GSE74767	6,109,506	**2,680,667**	1,139,307	907	1,546,768	741,857
GSM1932515	E07_MS03T58	PreL_TE	29	E07	whole embryo	121205ICSI#1day7	male	GSE74767	4,678,468	**2,160,704**	1,101,343	373	952,714	463,334
GSM1932517	E07_MS03T60	PreL_TE	30	E07	whole embryo	121205ICSI#1day7	male	GSE74767	6,956,412	**2,726,988**	1,786,233	632	1,727,298	715,261
GSM1932522	E07_MS08T45	PreL_TE	31	E07	whole embryo	130319ICSI#1day7	male	GSE74767	5,740,510	**2,232,954**	1,483,724	2,228	1,475,049	546,555
GSM1932521	E07_MS08T44	PreL_TE	32	E07	whole embryo	130319ICSI#1day7	male	GSE74767	4,781,234	**2,108,623**	902,393	1,499	1,321,849	446,870
GSM1932519	E07_MS03T67	PreL_TE	33	E07	whole embryo	130319ICSI#1day7	male	GSE74767	4,151,770	**1,584,429**	946,058	666	1,216,838	403,779
GSM1932520	E07_MS03T68	PreL_TE	34	E07	whole embryo	130319ICSI#1day7	male	GSE74767	4,716,991	**2,087,621**	902,820	1,142	1,271,443	453,965
GSM1932523	E07_MS08T46	PreL_TE	35	E07	whole embryo	130319ICSI#1day7	male	GSE74767	4,890,708	**2,248,541**	822,944	1,319	1,431,515	386,389
GSM1932540	E08_MS02T69	PreL_TE	36	E08	whole embryo	130220ICSI#11day8	male	GSE74767	3,924,513	**1,996,844**	712,726	2,113	802,376	410,454
GSM1932545	E08_MS02T75	PreL_TE	37	E08	whole embryo	130220ICSI#11day8	male	GSE74767	4,022,342	**1,975,185**	905,004	2,377	704,341	435,435
GSM1932542	E08_MS02T72	PreL_TE	38	E08	whole embryo	130220ICSI#11day8	male	GSE74767	3,946,813	**1,548,626**	1,175,149	694	748,307	474,037
GSM1932537	E08_MS02T62	PreL_TE	39	E08	whole embryo	130220ICSI#11day8	male	GSE74767	3,569,030	**1,682,482**	703,577	1,377	713,905	467,689
GSM1932544	E08_MS02T74	PreL_TE	40	E08	whole embryo	130220ICSI#11day8	male	GSE74767	4,197,156	**2,277,155**	750,534	1,742	734,496	433,229
GSM1932546	E08_MS02T78	PreL_TE	41	E08	whole embryo	130220ICSI#11day8	male	GSE74767	3,321,507	**1,630,392**	725,159	936	600,705	364,315
GSM1932548	E08_MS02T82	PreL_TE	42	E08	whole embryo	130220ICSI#11day8	male	GSE74767	3,708,067	**1,832,230**	742,293	1,174	734,749	397,621
GSM1932549	E08_MS02T83	PreL_TE	43	E08	whole embryo	130220ICSI#11day8	male	GSE74767	3,339,408	**1,811,022**	606,102	727	588,348	333,209
GSM1932536	E08_MS02T61	PreL_TE	44	E08	whole embryo	130220ICSI#11day8	male	GSE74767	3,166,483	**1,772,765**	568,943	1,146	472,607	351,022
GSM1932538	E08_MS02T64	PreL_TE	45	E08	whole embryo	130220ICSI#11day8	male	GSE74767	3,876,847	**2,034,822**	634,940	1,007	737,731	468,347
GSM1932539	E08_MS02T65	PreL_TE	46	E08	whole embryo	130220ICSI#11day8	male	GSE74767	4,026,786	**2,178,907**	759,252	1,170	695,027	392,430
GSM1932547	E08_MS02T79	PreL_TE	47	E08	whole embryo	130220ICSI#11day8	male	GSE74767	3,433,160	**1,861,980**	604,684	767	622,869	342,860
GSM1932556	E08_MS02T95	PreL_TE	48	E08	whole embryo	130220ICSI#11day8	male	GSE74767	3,784,209	**2,057,143**	648,876	1,243	643,604	433,343
GSM1932555	E08_MS02T94	PreL_TE	49	E08	whole embryo	130220ICSI#11day8	male	GSE74767	3,620,267	**2,008,743**	601,331	635	629,385	380,173
GSM1932557	E08_MS02T96	PreL_TE	50	E08	whole embryo	130220ICSI#11day8	male	GSE74767	1,435,755	**876,743**	219,031	452	195,689	143,840
GSM1932534	E08_MS02T58	PreL_TE	51	E08	whole embryo	130220ICSI#11day8	male	GSE74767	3,538,090	**1,978,308**	566,088	1,469	611,554	380,671
GSM1932550	E08_MS02T86	PreL_TE	52	E08	whole embryo	130220ICSI#11day8	male	GSE74767	3,581,807	**1,891,030**	531,717	439	695,572	463,049
GSM1932541	E08_MS02T70	PreL_TE	53	E08	whole embryo	130220ICSI#11day8	male	GSE74767	3,448,892	**1,883,198**	627,628	930	596,782	340,354
GSM1932543	E08_MS02T73	PreL_TE	54	E08	whole embryo	130220ICSI#11day8	male	GSE74767	3,143,604	**1,664,583**	575,809	1,134	583,512	318,566
GSM1932535	E08_MS02T60	PreL_TE	55	E08	whole embryo	130220ICSI#11day8	male	GSE74767	3,515,803	**1,705,807**	751,137	1,606	624,196	433,057
GSM1932551	E08_MS02T87	PreL_TE	56	E08	whole embryo	130220ICSI#11day8	male	GSE74767	3,391,374	**1,794,959**	653,114	941	551,402	390,958
GSM1932553	E08_MS02T90	PreL_TE	57	E08	whole embryo	130220ICSI#11day8	male	GSE74767	3,930,689	**1,974,787**	792,200	409	680,532	482,761
GSM1932552	E08_MS02T88	PreL_TE	58	E08	whole embryo	130220ICSI#11day8	male	GSE74767	3,312,599	**1,599,652**	605,333	540	701,419	405,655
GSM1932554	E08_MS02T91	PreL_TE	59	E08	whole embryo	130220ICSI#11day8	male	GSE74767	2,856,778	**1,504,603**	567,425	952	455,171	328,627
GSM1932516	E07_MS03T59	PreL_TE	60	E07	whole embryo	121205ICSI#1day7	male	GSE74767	3,548,957	**1,432,388**	703,706	1,092	1,048,476	363,295
GSM1932564	E09_MS03T90	PreL_TE	61	E09	whole embryo	121212ICSI#2day9	female	GSE74767	5,709,106	**2,489,234**	1,016,452	1,081	1,658,458	543,881
GSM1932563	E09_MS03T87	PreL_TE	62	E09	whole embryo	121212ICSI#2day9	female	GSE74767	4,017,390	**1,754,220**	721,675	450	1,153,287	387,758
GSM1932565	E09_MS03T94	PreL_TE	63	E09	whole embryo	121212ICSI#2day9	female	GSE74767	5,352,204	**2,142,251**	929,021	1,406	1,768,607	510,919
GSM1932278	E08_MS02T23	Hypoblast	64	E08	whole embryo	121107ICSI13_day8	male	GSE74767	6,253,800	**3,311,662**	1,150,934	2,685	1,136,944	651,575
GSM1932265	E07_MS03T72	Hypoblast	65	E07	whole embryo	130319ICSI#1day7	male	GSE74767	4,116,751	**1,472,479**	1,003,075	2,241	1,233,305	405,651
GSM1932270	E08_MS02T12	Hypoblast	66	E08	whole embryo	121107ICSI13_day8	male	GSE74767	8,297,727	**2,819,205**	3,729,548	3,238	912,304	833,432
GSM1932275	E08_MS02T20	Hypoblast	67	E08	whole embryo	121107ICSI13_day8	male	GSE74767	7,579,424	**2,832,280**	2,976,128	2,346	986,177	782,493
GSM1932304	E09_MS03T78	Hypoblast	68	E09	whole embryo	120725ICSIday9	male	GSE74767	6,213,540	**2,610,118**	1,362,017	3,049	1,458,223	780,133
GSM1932258	E07_MS03T54	Hypoblast	69	E07	whole embryo	121205ICSI#1day7	male	GSE74767	1,672,175	**679,947**	330,500	424	487,044	174,260
GSM1932264	E07_MS03T71	Hypoblast	70	E07	whole embryo	130319ICSI#1day7	male	GSE74767	4,374,061	**1,858,315**	1,004,343	1,253	1,108,676	401,474
GSM1932283	E08_MS02T35	Hypoblast	71	E08	whole embryo	121107ICSI13_day8	male	GSE74767	5,559,037	**2,957,509**	1,240,210	2,896	824,688	533,734
GSM1932309	E09_MS03T93	Hypoblast	72	E09	whole embryo	121212ICSI#2day9	female	GSE74767	4,047,218	**1,727,535**	611,741	1,057	1,318,679	388,206
GSM1932301	E08_MS02T80	Hypoblast	73	E08	whole embryo	130220ICSI#11day8	male	GSE74767	2,928,563	**1,546,076**	574,730	1,521	518,755	287,481
GSM1932294	E08_MS02T56	Hypoblast	74	E08	whole embryo	121212ICSI#11day8	male	GSE74767	3,703,150	**1,801,845**	679,234	752	817,974	403,345
GSM1932307	E09_MS03T88	Hypoblast	75	E09	whole embryo	121212ICSI#2day9	female	GSE74767	4,510,417	**1,802,845**	887,587	269	1,351,497	468,219
GSM1932271	E08_MS02T13	Hypoblast	76	E08	whole embryo	121107ICSI13_day8	male	GSE74767	5,456,909	**2,489,880**	1,597,628	2,722	805,899	560,780
GSM1932273	E08_MS02T15	Hypoblast	77	E08	whole embryo	121107ICSI13_day8	male	GSE74767	4,900,508	**2,317,010**	1,309,031	2,093	774,212	498,162
GSM1932289	E08_MS02T46	Hypoblast	78	E08	whole embryo	121212ICSI#11day8	male	GSE74767	2,740,751	**1,443,739**	271,601	2,550	760,299	262,562
GSM1932297	E08_MS02T67	Hypoblast	79	E08	whole embryo	130220ICSI#11day8	male	GSE74767	3,038,262	**1,317,612**	555,726	800	822,919	341,205
GSM1932299	E08_MS02T71	Hypoblast	80	E08	whole embryo	130220ICSI#11day8	male	GSE74767	4,334,836	**2,296,508**	826,140	2,254	776,926	433,008
GSM1932296	E08_MS02T66	Hypoblast	81	E08	whole embryo	130220ICSI#11day8	male	GSE74767	3,606,216	**1,761,448**	743,823	1,830	688,599	410,516
GSM1932295	E08_MS02T63	Hypoblast	82	E08	whole embryo	130220ICSI#11day8	male	GSE74767	3,825,054	**1,888,277**	675,624	1,339	803,707	456,107
GSM1932298	E08_MS02T68	Hypoblast	83	E08	whole embryo	130220ICSI#11day8	male	GSE74767	4,698,998	**2,448,369**	927,116	1,086	830,291	492,136
GSM1932300	E08_MS02T77	Hypoblast	84	E08	whole embryo	130220ICSI#11day8	male	GSE74767	3,503,384	**1,762,556**	787,325	1,375	589,409	362,719
GSM1932292	E08_MS02T53	Hypoblast	85	E08	whole embryo	121212ICSI#11day8	male	GSE74767	3,064,629	**1,288,864**	984,174	802	455,389	335,400
GSM1932286	E08_MS02T40	Hypoblast	86	E08	whole embryo	121212ICSI#11day8	male	GSE74767	2,999,552	**1,314,476**	543,353	684	813,435	327,604
GSM1932266	E07_MS08T43	Hypoblast	87	E07	whole embryo	121205ICSI#1day7	male	GSE74767	5,884,380	**2,274,787**	1,269,340	2,020	1,710,448	627,785
GSM1932261	E07_MS03T61	Hypoblast	88	E07	whole embryo	121205ICSI#1day7	male	GSE74767	5,050,434	**2,679,033**	830,810	1,108	1,063,682	475,801
GSM1932262	E07_MS03T62	Hypoblast	89	E07	whole embryo	121205ICSI#1day7	male	GSE74767	3,737,959	**1,253,448**	454,121	696	1,641,288	388,406
GSM1932257	E07_MS03T53	Hypoblast	90	E07	whole embryo	121205ICSI#1day7	male	GSE74767	3,247,062	**1,380,594**	561,520	978	985,829	318,141
GSM1932260	E07_MS03T57	Hypoblast	91	E07	whole embryo	121205ICSI#1day7	male	GSE74767	4,228,060	**1,649,137**	735,846	837	1,416,930	425,310
GSM1932263	E07_MS03T64	Hypoblast	92	E07	whole embryo	121205ICSI#1day7	male	GSE74767	5,658,536	**2,448,635**	869,028	4,642	1,783,411	552,820
GSM1932293	E08_MS02T54	Hypoblast	93	E08	whole embryo	121212ICSI#11day8	male	GSE74767	2,121,073	**1,021,847**	521,055	440	360,424	217,307
GSM1932305	E09_MS03T80	Hypoblast	94	E09	whole embryo	120725ICSIday9	male	GSE74767	5,386,009	**2,755,211**	938,497	1,999	1,122,425	567,877
GSM1932306	E09_MS03T81	Hypoblast	95	E09	whole embryo	120725ICSIday9	male	GSE74767	6,122,695	**2,982,307**	1,167,681	1,772	1,295,494	675,441
GSM1932290	E08_MS02T49	Hypoblast	96	E08	whole embryo	121212ICSI#11day8	male	GSE74767	2,382,124	**1,168,237**	433,819	603	518,283	261,182
GSM1932259	E07_MS03T56	Hypoblast	97	E07	whole embryo	121205ICSI#1day7	male	GSE74767	5,436,682	**2,065,629**	723,122	1,238	2,102,206	544,487
GSM1932302	E08_MS02T81	Hypoblast	98	E08	whole embryo	130220ICSI#11day8	male	GSE74767	3,168,404	**1,534,046**	587,887	759	702,026	343,686
GSM1932288	E08_MS02T42	Hypoblast	99	E08	whole embryo	121212ICSI#11day8	male	GSE74767	3,794,210	**1,757,710**	635,730	641	1,004,093	396,036
GSM1932284	E08_MS02T37	Hypoblast	100	E08	whole embryo	121212ICSI#11day8	male	GSE74767	3,543,589	**1,583,178**	726,407	480	862,831	370,693
GSM1932291	E08_MS02T50	Hypoblast	101	E08	whole embryo	121212ICSI#11day8	male	GSE74767	4,422,671	**2,243,836**	900,940	379	812,274	465,242
GSM1932303	E08_MS03T02	Hypoblast	102	E08	whole embryo	121212ICSI#11day8	male	GSE74767	4,924,109	**1,686,073**	995,253	1,376	1,750,956	490,451
GSM1932285	E08_MS02T39	Hypoblast	103	E08	whole embryo	121212ICSI#11day8	male	GSE74767	2,492,058	**1,125,935**	504,315	525	595,034	266,249
GSM1932287	E08_MS02T41	Hypoblast	104	E08	whole embryo	121212ICSI#11day8	male	GSE74767	2,784,628	**1,064,290**	416,610	762	981,362	321,604
GSM1932308	E09_MS03T89	Hypoblast	105	E09	whole embryo	121212ICSI#2day9	female	GSE74767	4,556,388	**1,787,184**	627,169	835	1,675,217	465,983
GSM1932310	E09_MS03T95	Hypoblast	106	E09	whole embryo	121212ICSI#2day9	female	GSE74767	3,987,985	**1,618,676**	529,743	1,452	1,445,851	392,263
GSM1932282	E08_MS02T32	Hypoblast	107	E08	whole embryo	121107ICSI13_day8	male	GSE74767	5,385,527	**2,641,107**	1,362,869	3,915	824,154	553,482
GSM1932277	E08_MS02T22	Hypoblast	108	E08	whole embryo	121107ICSI13_day8	male	GSE74767	7,441,517	**3,341,675**	2,087,441	3,203	1,237,182	772,016
GSM1932280	E08_MS02T28	Hypoblast	109	E08	whole embryo	121107ICSI13_day8	male	GSE74767	5,746,208	**2,867,676**	1,414,059	1,479	897,868	565,126
GSM1932281	E08_MS02T29	Hypoblast	110	E08	whole embryo	121107ICSI13_day8	male	GSE74767	5,122,182	**2,500,056**	1,341,557	1,863	788,259	490,447
GSM1932269	E08_MS02T09	Hypoblast	111	E08	whole embryo	121107ICSI13_day8	male	GSE74767	5,603,782	**2,774,200**	1,514,425	2,196	777,795	535,166
GSM1932267	E08_MS02T03	Hypoblast	112	E08	whole embryo	121107ICSI13_day8	male	GSE74767	5,466,255	**2,874,759**	1,207,637	2,313	863,322	518,224
GSM1932279	E08_MS02T26	Hypoblast	113	E08	whole embryo	121107ICSI13_day8	male	GSE74767	5,794,104	**3,066,464**	1,333,685	2,278	857,213	534,464
GSM1932268	E08_MS02T08	Hypoblast	114	E08	whole embryo	121107ICSI13_day8	male	GSE74767	5,182,804	**2,283,877**	1,437,874	1,996	901,736	557,321
GSM1932276	E08_MS02T21	Hypoblast	115	E08	whole embryo	121107ICSI13_day8	male	GSE74767	5,453,815	**3,079,438**	1,009,470	1,872	852,175	510,860
GSM1932272	E08_MS02T14	Hypoblast	116	E08	whole embryo	121107ICSI13_day8	male	GSE74767	7,097,619	**3,522,049**	1,840,343	1,871	1,039,334	694,022
GSM1932274	E08_MS02T18	Hypoblast	117	E08	whole embryo	121107ICSI13_day8	male	GSE74767	8,340,331	**4,630,817**	1,490,805	2,366	1,382,157	834,186
GSM1932500	E06_MS03T30	PreE_TE	118	E06	whole embryo	130227ICSI#1day6	female	GSE74767	6,944,413	**2,754,186**	1,623,766	568	1,723,852	842,041
GSM1932328	E06_MS03T38	PreE_TE	119	E06	whole embryo	130306ICSI#7day6	female	GSE74767	5,491,733	**1,859,417**	1,334,132	370	1,530,297	767,517
GSM1932501	E06_MS68T13	PreE_TE	120	E06	whole embryo	130227ICSI#1day6	female	GSE74767	4,122,463	**1,641,710**	928,557	879	1,104,197	447,120
GSM1932496	E06_MS03T24	PreE_TE	121	E06	whole embryo	130227ICSI#1day6	female	GSE74767	6,827,318	**2,640,660**	1,514,237	1,089	1,889,713	781,619
GSM1932503	E06_MS68T16	PreE_TE	122	E06	whole embryo	130227ICSI#1day6	female	GSE74767	2,893,961	**1,093,097**	510,406	603	1,017,745	272,110
GSM1932493	E06_MS03T19	PreE_TE	123	E06	whole embryo	130227ICSI#1day6	female	GSE74767	3,330,196	**1,282,712**	704,022	580	971,107	371,775
GSM1932498	E06_MS03T27	PreE_TE	124	E06	whole embryo	130227ICSI#1day6	female	GSE74767	5,420,210	**2,280,662**	1,152,813	625	1,349,682	636,428
GSM1932497	E06_MS03T25	PreE_TE	125	E06	whole embryo	130227ICSI#1day6	female	GSE74767	4,714,281	**1,974,147**	1,015,941	694	1,166,128	557,371
GSM1932494	E06_MS03T21	PreE_TE	126	E06	whole embryo	130227ICSI#1day6	female	GSE74767	4,913,293	**2,089,458**	970,368	685	1,316,400	536,382
GSM1932491	E06_MS03T16	PreE_TE	127	E06	whole embryo	130227ICSI#1day6	female	GSE74767	6,277,089	**2,479,631**	1,251,603	1,074	1,796,953	747,828
GSM1932492	E06_MS03T17	PreE_TE	128	E06	whole embryo	130227ICSI#1day6	female	GSE74767	4,979,264	**2,199,305**	953,015	539	1,272,417	553,988
GSM1932502	E06_MS68T15	PreE_TE	129	E06	whole embryo	130227ICSI#1day6	female	GSE74767	2,897,604	**1,080,308**	741,179	621	735,034	340,462
GSM1932495	E06_MS03T22	PreE_TE	130	E06	whole embryo	130227ICSI#1day6	female	GSE74767	4,651,187	**1,704,550**	996,999	509	1,420,828	528,301
GSM1932499	E06_MS03T29	PreE_TE	131	E06	whole embryo	130227ICSI#1day6	female	GSE74767	5,978,956	**2,839,558**	1,019,870	736	1,544,425	574,367
GSM1932510	E07_MS03T46	PreE_TE	132	E07	whole embryo	121128ICSI#1day7	male	GSE74767	4,582,757	**1,027,348**	1,450,613	1,842	1,257,947	845,007
GSM1932508	E07_MS03T44	PreE_TE	133	E07	whole embryo	121128ICSI#1day7	male	GSE74767	5,353,604	**1,334,901**	2,017,416	926	1,277,041	723,320
GSM1932507	E07_MS03T43	PreE_TE	134	E07	whole embryo	121128ICSI#1day7	male	GSE74767	6,033,396	**2,176,475**	1,358,292	770	1,644,763	853,096
GSM1932506	E07_MS03T42	PreE_TE	135	E07	whole embryo	121128ICSI#1day7	male	GSE74767	4,791,070	**2,076,983**	875,287	1,258	1,292,582	544,960
GSM1932513	E07_MS03T49	PreE_TE	136	E07	whole embryo	121128ICSI#1day7	male	GSE74767	4,747,124	**1,973,432**	1,245,732	1,678	927,008	599,274
GSM1932509	E07_MS03T45	PreE_TE	137	E07	whole embryo	121128ICSI#1day7	male	GSE74767	4,843,458	**1,933,285**	1,120,766	683	1,203,336	585,388
GSM1932511	E07_MS03T47	PreE_TE	138	E07	whole embryo	121128ICSI#1day7	male	GSE74767	5,774,869	**2,221,827**	1,240,890	700	1,492,525	818,927
GSM1932505	E07_MS03T41	PreE_TE	139	E07	whole embryo	121128ICSI#1day7	male	GSE74767	5,950,583	**2,343,854**	1,517,510	700	1,396,080	692,439
GSM1932504	E07_MS03T40	PreE_TE	140	E07	whole embryo	121128ICSI#1day7	male	GSE74767	4,800,241	**2,176,400**	1,040,298	867	1,025,341	557,335
GSM1932512	E07_MS03T48	PreE_TE	141	E07	whole embryo	121128ICSI#1day7	male	GSE74767	5,530,186	**2,309,733**	1,096,898	900	1,387,157	735,498
GSM1932334	E06_MS68T06	ICM	142	E06	whole embryo	130206ICSI#1day6	male	GSE74767	2,942,999	**1,033,265**	654,289	214	1,024,403	230,828
GSM1932330	E06_MS68T02	ICM	143	E06	whole embryo	130206ICSI#1day6	male	GSE74767	3,662,545	**1,260,599**	854,612	112	1,264,635	282,587
GSM1932336	E06_MS68T08	ICM	144	E06	whole embryo	130206ICSI#1day6	male	GSE74767	1,718,888	**688,659**	381,073	135	507,758	141,263
GSM1932340	E06_MS68T12	ICM	145	E06	whole embryo	130206ICSI#1day6	male	GSE74767	5,716,490	**2,156,704**	1,214,981	223	1,852,795	491,787
GSM1932338	E06_MS68T10	ICM	146	E06	whole embryo	130206ICSI#1day6	male	GSE74767	3,036,423	**1,127,865**	496,022	106	1,172,828	239,602
GSM1932311	E06_MS03T03	ICM	147	E06	whole embryo	130206ICSI#1day6	male	GSE74767	4,310,758	**1,732,807**	933,992	90	1,214,988	428,881
GSM1932331	E06_MS68T03	ICM	148	E06	whole embryo	130206ICSI#1day6	male	GSE74767	2,995,292	**1,236,380**	587,957	106	939,372	231,477
GSM1932332	E06_MS68T04	ICM	149	E06	whole embryo	130206ICSI#1day6	male	GSE74767	3,590,300	**1,303,501**	674,612	120	1,352,639	259,428
GSM1932327	E06_MS03T34	ICM	150	E06	whole embryo	130306ICSI#7day6	female	GSE74767	3,667,894	**1,784,545**	774,838	349	716,505	391,657
GSM1932329	E06_MS68T01	ICM	151	E06	whole embryo	130206ICSI#1day6	male	GSE74767	3,035,025	**1,077,323**	859,981	199	834,737	262,785
GSM1932339	E06_MS68T11	ICM	152	E06	whole embryo	130206ICSI#1day6	male	GSE74767	2,261,084	**947,479**	494,808	67	633,725	185,005
GSM1932314	E06_MS03T06	ICM	153	E06	whole embryo	130206ICSI#1day6	male	GSE74767	5,871,201	**2,615,551**	1,173,228	121	1,520,325	561,976
GSM1932333	E06_MS68T05	ICM	154	E06	whole embryo	130206ICSI#1day6	male	GSE74767	3,128,282	**1,180,113**	915,981	93	757,868	274,227
GSM1932337	E06_MS68T09	ICM	155	E06	whole embryo	130206ICSI#1day6	male	GSE74767	2,904,099	**934,305**	630,137	189	1,121,586	217,882
GSM1932321	E06_MS03T14	ICM	156	E06	whole embryo	130206ICSI#1day6	male	GSE74767	8,533,262	**3,753,526**	1,959,436	409	2,024,870	795,021
GSM1932318	E06_MS03T11	ICM	157	E06	whole embryo	130206ICSI#1day6	male	GSE74767	5,235,517	**1,923,956**	1,385,484	165	1,401,869	524,043
GSM1932335	E06_MS68T07	ICM	158	E06	whole embryo	130206ICSI#1day6	male	GSE74767	2,175,030	**791,535**	470,022	93	734,063	179,317
GSM1932319	E06_MS03T12	ICM	159	E06	whole embryo	130206ICSI#1day6	male	GSE74767	4,737,642	**1,935,172**	1,217,903	389	1,107,964	476,214
GSM1932320	E06_MS03T13	ICM	160	E06	whole embryo	130206ICSI#1day6	male	GSE74767	6,715,658	**2,623,120**	1,568,355	217	1,843,399	680,567
GSM1932312	E06_MS03T04	ICM	161	E06	whole embryo	130206ICSI#1day6	male	GSE74767	4,626,086	**1,596,212**	1,343,358	263	1,214,874	471,379
GSM1932313	E06_MS03T05	ICM	162	E06	whole embryo	130206ICSI#1day6	male	GSE74767	5,933,397	**2,005,358**	1,431,224	247	1,874,416	622,152
GSM1932316	E06_MS03T08	ICM	163	E06	whole embryo	130206ICSI#1day6	male	GSE74767	4,894,876	**1,677,663**	1,430,825	171	1,274,381	511,836
GSM1932315	E06_MS03T07	ICM	164	E06	whole embryo	130206ICSI#1day6	male	GSE74767	5,905,135	**2,179,900**	1,646,276	188	1,474,665	604,106
GSM1932317	E06_MS03T10	ICM	165	E06	whole embryo	130206ICSI#1day6	male	GSE74767	5,598,031	**2,064,974**	1,490,856	115	1,463,758	578,328
GSM1932326	E06_MS03T28	ICM	166	E06	whole embryo	130227ICSI#1day6	female	GSE74767	5,825,270	**1,864,102**	1,781,052	553	1,395,959	783,604
GSM1932325	E06_MS03T26	ICM	167	E06	whole embryo	130227ICSI#1day6	female	GSE74767	7,305,783	**2,903,429**	1,666,231	1,755	1,939,730	794,638
GSM1932324	E06_MS03T23	ICM	168	E06	whole embryo	130227ICSI#1day6	female	GSE74767	5,453,926	**1,952,589**	1,494,436	741	1,324,192	681,968
GSM1932322	E06_MS03T15	ICM	169	E06	whole embryo	130227ICSI#1day6	female	GSE74767	7,115,508	**1,852,598**	2,569,191	615	1,637,754	1,055,350
GSM1932323	E06_MS03T20	ICM	170	E06	whole embryo	130227ICSI#1day6	female	GSE74767	3,383,309	**1,414,237**	691,234	647	912,830	364,361
GSM1932470	E08_MS02T36	Pre_EPI	171	E08	whole embryo	121107ICSI13_day8	male	GSE74767	5,030,935	**2,503,909**	1,361,791	2,032	706,360	456,843
GSM1932464	E08_MS02T05	Pre_EPI	172	E08	whole embryo	121107ICSI13_day8	male	GSE74767	6,383,922	**3,136,083**	1,587,943	2,845	1,031,883	625,168
GSM1932466	E08_MS02T16	Pre_EPI	173	E08	whole embryo	121107ICSI13_day8	male	GSE74767	6,462,396	**3,215,522**	1,511,766	1,791	1,073,194	660,123
GSM1932467	E08_MS02T17	Pre_EPI	174	E08	whole embryo	121107ICSI13_day8	male	GSE74767	6,095,474	**2,924,677**	1,593,399	1,447	972,598	603,353
GSM1932469	E08_MS02T31	Pre_EPI	175	E08	whole embryo	121107ICSI13_day8	male	GSE74767	5,540,759	**2,865,042**	1,229,822	1,746	909,487	534,662
GSM1932468	E08_MS02T30	Pre_EPI	176	E08	whole embryo	121107ICSI13_day8	male	GSE74767	6,222,423	**3,052,728**	1,554,247	1,748	986,756	626,944
GSM1932463	E08_MS02T02	Pre_EPI	177	E08	whole embryo	121107ICSI13_day8	male	GSE74767	6,997,596	**3,521,486**	1,715,673	2,519	1,085,482	672,436
GSM1932465	E08_MS02T06	Pre_EPI	178	E08	whole embryo	121107ICSI13_day8	male	GSE74767	8,210,584	**3,855,825**	2,311,515	1,033	1,244,790	797,421
GSM1932487	E09_MS03T82	Pre_EPI	179	E09	whole embryo	120725ICSIday9	male	GSE74767	5,911,096	**2,630,317**	1,144,709	3,174	1,454,901	677,995
GSM1932489	E09_MS03T85	Pre_EPI	180	E09	whole embryo	120725ICSIday9	male	GSE74767	5,756,563	**2,151,618**	1,595,354	1,394	1,264,180	744,017
GSM1932481	E08_MS02T85	Pre_EPI	181	E08	whole embryo	130220ICSI#11day8	male	GSE74767	3,226,087	**1,652,615**	614,776	756	622,276	335,664
GSM1932483	E08_MS02T92	Pre_EPI	182	E08	whole embryo	130220ICSI#11day8	male	GSE74767	3,446,447	**1,870,723**	583,028	775	621,742	370,179
GSM1932480	E08_MS02T59	Pre_EPI	183	E08	whole embryo	130220ICSI#11day8	male	GSE74767	3,975,653	**2,175,367**	667,993	1,021	689,611	441,661
GSM1932484	E08_MS02T93	Pre_EPI	184	E08	whole embryo	130220ICSI#11day8	male	GSE74767	3,290,342	**1,760,659**	577,301	742	594,363	357,277
GSM1932488	E09_MS03T83	Pre_EPI	185	E09	whole embryo	120725ICSIday9	male	GSE74767	6,247,104	**2,850,546**	1,164,757	1,409	1,506,375	724,017
GSM1932486	E09_MS03T79	Pre_EPI	186	E09	whole embryo	120725ICSIday9	male	GSE74767	4,696,577	**2,037,773**	980,869	1,115	1,122,284	554,536
GSM1932471	E08_MS02T38	Pre_EPI	187	E08	whole embryo	121212ICSI#11day8	male	GSE74767	3,953,616	**1,820,660**	785,167	735	922,793	424,261
GSM1932482	E08_MS02T89	Pre_EPI	188	E08	whole embryo	130220ICSI#11day8	male	GSE74767	4,143,227	**2,091,560**	846,679	477	709,076	495,435
GSM1932485	E08_MS03T01	Pre_EPI	189	E08	whole embryo	130220ICSI#11day8	male	GSE74767	5,560,690	**2,130,831**	1,435,800	916	1,316,457	676,686
GSM1932459	E07_MS03T70	Pre_EPI	190	E07	whole embryo	130319ICSI#1day7	male	GSE74767	5,017,381	**1,908,365**	1,113,926	1,317	1,487,919	505,854
GSM1932462	E08_MS02T01	Pre_EPI	191	E08	whole embryo	121107ICSI13_day8	male	GSE74767	6,961,614	**2,885,495**	2,328,845	2,925	1,027,889	716,460
GSM1932490	E09_MS03T91	Pre_EPI	192	E09	whole embryo	121212ICSI#2day9	female	GSE74767	3,746,140	**1,502,322**	753,474	876	1,092,474	396,994
GSM1932457	E07_MS03T52	Pre_EPI	193	E07	whole embryo	121205ICSI#1day7	male	GSE74767	3,913,000	**1,296,786**	582,916	640	1,601,122	431,536
GSM1932458	E07_MS03T65	Pre_EPI	194	E07	whole embryo	121205ICSI#1day7	male	GSE74767	3,326,435	**1,206,204**	477,638	707	1,312,096	329,790
GSM1932474	E08_MS02T45	Pre_EPI	195	E08	whole embryo	121212ICSI#11day8	male	GSE74767	2,633,093	**809,519**	807,506	1,038	699,159	315,871
GSM1932476	E08_MS02T48	Pre_EPI	196	E08	whole embryo	121212ICSI#11day8	male	GSE74767	4,618,458	**2,267,311**	855,484	1,393	1,036,888	457,382
GSM1932478	E08_MS02T55	Pre_EPI	197	E08	whole embryo	121212ICSI#11day8	male	GSE74767	2,702,684	**1,369,998**	520,748	843	534,669	276,426
GSM1932460	E07_MS08T41	Pre_EPI	198	E07	whole embryo	121205ICSI#1day7	male	GSE74767	4,487,291	**1,685,809**	905,915	758	1,431,994	462,815
GSM1932472	E08_MS02T43	Pre_EPI	199	E08	whole embryo	121212ICSI#11day8	male	GSE74767	3,663,627	**1,150,153**	1,098,507	822	986,098	428,047
GSM1932475	E08_MS02T47	Pre_EPI	200	E08	whole embryo	121212ICSI#11day8	male	GSE74767	3,624,214	**1,455,132**	641,544	779	1,139,473	387,286
GSM1932461	E07_MS08T42	Pre_EPI	201	E07	whole embryo	121205ICSI#1day7	male	GSE74767	4,966,321	**1,898,568**	786,661	693	1,794,445	485,954
GSM1932473	E08_MS02T44	Pre_EPI	202	E08	whole embryo	121212ICSI#11day8	male	GSE74767	3,663,855	**1,572,060**	749,161	561	977,792	364,281
GSM1932477	E08_MS02T52	Pre_EPI	203	E08	whole embryo	121212ICSI#11day8	male	GSE74767	3,431,679	**1,681,422**	587,613	878	787,337	374,429
GSM1932479	E08_MS02T57	Pre_EPI	204	E08	whole embryo	121212ICSI#11day8	male	GSE74767	3,634,685	**1,821,962**	709,497	913	708,169	394,144
GSM1932383	E14_MS08T52	PostE_EPI	205	E14	around embryonic disk	140401ICSIday14	male	GSE74767	5,018,860	**2,310,794**	849,358	41,034	1,223,490	594,184
GSM1932385	E14_MS08T54	PostE_EPI	206	E14	around embryonic disk	140401ICSIday14	male	GSE74767	3,654,049	**1,639,829**	687,972	28,356	900,880	397,012
GSM1932390	E14_MS10T62	PostE_EPI	207	E14	around embryonic disk	140401ICSIday14	male	GSE74767	3,267,201	**1,313,405**	573,627	18,403	970,348	391,418
GSM1932380	E14_MS08T49	PostE_EPI	208	E14	around embryonic disk	140401ICSIday14	male	GSE74767	4,131,755	**2,061,105**	660,675	38,247	938,004	433,724
GSM1932382	E14_MS08T51	PostE_EPI	209	E14	around embryonic disk	140401ICSIday14	male	GSE74767	4,649,292	**1,929,765**	944,233	29,120	1,241,423	504,751
GSM1932391	E14_MS10T63	PostE_EPI	210	E14	around embryonic disk	140401ICSIday14	male	GSE74767	5,357,391	**1,646,750**	1,206,422	33,881	1,752,267	718,071
GSM1932389	E14_MS10T61	PostE_EPI	211	E14	around embryonic disk	140401ICSIday14	male	GSE74767	3,452,452	**1,470,294**	543,136	14,227	1,075,532	349,263
GSM1932379	E14_MS08T48	PostE_EPI	212	E14	around embryonic disk	140401ICSIday14	male	GSE74767	5,380,776	**2,294,964**	1,131,044	22,573	1,313,719	618,476
GSM1932386	E14_MS08T55	PostE_EPI	213	E14	around embryonic disk	140401ICSIday14	male	GSE74767	3,504,639	**1,512,957**	645,824	23,157	910,255	412,446
GSM1932384	E14_MS08T53	PostE_EPI	214	E14	around embryonic disk	140401ICSIday14	male	GSE74767	4,143,768	**2,084,074**	848,006	12,178	737,024	462,486
GSM1932387	E14_MS08T56	PostE_EPI	215	E14	around embryonic disk	140401ICSIday14	male	GSE74767	4,370,587	**1,327,520**	784,876	20,863	1,643,860	593,468
GSM1932381	E14_MS08T50	PostE_EPI	216	E14	around embryonic disk	140401ICSIday14	male	GSE74767	5,381,220	**2,042,304**	786,798	30,413	1,899,262	622,443
GSM1932388	E14_MS10T60	PostE_EPI	217	E14	around embryonic disk	140401ICSIday14	male	GSE74767	3,406,261	**1,130,970**	398,835	31,279	1,493,031	352,146
GSM1932352	E13_MS10T34	PostE_EPI	218	E13	around embryonic disk	140812ICSId13A	male	GSE74767	3,631,183	**1,310,399**	574,706	25,917	1,339,825	380,336
GSM1932372	E13_MS61T52	PostE_EPI	219	E13	around embryonic disk	140812ICSId13A	male	GSE74767	4,888,568	**2,168,287**	853,587	42,138	1,281,081	543,475
GSM1932356	E13_MS10T39	PostE_EPI	220	E13	around embryonic disk	140812ICSId13A	male	GSE74767	3,381,261	**1,205,866**	498,956	21,784	1,280,474	374,181
GSM1932374	E13_MS61T55	PostE_EPI	221	E13	around embryonic disk	140812ICSId13A	male	GSE74767	4,153,188	**2,039,724**	640,879	28,133	1,040,898	403,554
GSM1932366	E13_MS10T58	PostE_EPI	222	E13	around embryonic disk	140812ICSId13A	male	GSE74767	3,661,460	**1,244,055**	479,841	21,986	1,522,610	392,968
GSM1932361	E13_MS10T52	PostE_EPI	223	E13	around embryonic disk	140812ICSId13A	male	GSE74767	6,969,917	**2,243,634**	828,708	36,783	3,079,261	781,531
GSM1932355	E13_MS10T37	PostE_EPI	224	E13	around embryonic disk	140812ICSId13A	male	GSE74767	4,732,417	**1,692,455**	631,569	19,068	1,872,668	516,657
GSM1932357	E13_MS10T40	PostE_EPI	225	E13	around embryonic disk	140812ICSId13A	male	GSE74767	4,253,097	**1,240,030**	523,745	17,892	2,009,047	462,383
GSM1932377	E13_MS61T58	PostE_EPI	226	E13	around embryonic disk	140812ICSId13A	male	GSE74767	5,046,660	**2,538,779**	817,034	29,195	1,163,910	497,742
GSM1932367	E13_MS10T59	PostE_EPI	227	E13	around embryonic disk	140812ICSId13A	male	GSE74767	5,327,403	**2,036,297**	688,892	21,098	1,953,234	627,882
GSM1932358	E13_MS10T43	PostE_EPI	228	E13	around embryonic disk	140812ICSId13A	male	GSE74767	4,064,616	**1,109,244**	479,640	13,115	1,991,049	471,568
GSM1932364	E13_MS10T56	PostE_EPI	229	E13	around embryonic disk	140812ICSId13A	male	GSE74767	5,580,776	**2,294,746**	723,015	25,072	1,895,902	642,041
GSM1932368	E13_MS13T92	PostE_EPI	230	E13	around embryonic disk	140812ICSId13A	male	GSE74767	4,886,895	**1,833,952**	887,046	29,651	1,518,995	617,251
GSM1932370	E13_MS61T47	PostE_EPI	231	E13	around embryonic disk	140812ICSId13A	male	GSE74767	7,680,274	**3,126,791**	1,302,423	57,941	2,345,974	847,145
GSM1932376	E13_MS61T57	PostE_EPI	232	E13	around embryonic disk	140812ICSId13A	male	GSE74767	5,109,115	**2,058,620**	720,968	31,317	1,740,321	557,889
GSM1932360	E13_MS10T51	PostE_EPI	233	E13	around embryonic disk	140812ICSId13A	male	GSE74767	3,820,780	**1,379,623**	543,875	25,280	1,455,043	416,959
GSM1932354	E13_MS10T36	PostE_EPI	234	E13	around embryonic disk	140812ICSId13A	male	GSE74767	4,611,068	**1,704,528**	793,149	33,585	1,531,220	548,586
GSM1932365	E13_MS10T57	PostE_EPI	235	E13	around embryonic disk	140812ICSId13A	male	GSE74767	5,477,540	**1,720,551**	664,069	24,273	2,454,522	614,125
GSM1932371	E13_MS61T48	PostE_EPI	236	E13	around embryonic disk	140812ICSId13A	male	GSE74767	6,600,195	**2,670,987**	1,224,134	62,909	1,811,115	831,050
GSM1932375	E13_MS61T56	PostE_EPI	237	E13	around embryonic disk	140812ICSId13A	male	GSE74767	4,705,431	**2,206,137**	820,721	44,683	1,131,729	502,161
GSM1932362	E13_MS10T54	PostE_EPI	238	E13	around embryonic disk	140812ICSId13A	male	GSE74767	2,960,331	**916,683**	326,728	14,808	1,477,832	224,280
GSM1932378	E13_MS61T59	PostE_EPI	239	E13	around embryonic disk	140812ICSId13A	male	GSE74767	4,971,556	**1,948,851**	751,203	37,700	1,665,787	568,015
GSM1932369	E13_MS61T46	PostE_EPI	240	E13	around embryonic disk	140812ICSId13A	male	GSE74767	5,407,134	**2,239,901**	888,867	56,532	1,728,923	492,911
GSM1932373	E13_MS61T53	PostE_EPI	241	E13	around embryonic disk	140812ICSId13A	male	GSE74767	5,134,799	**1,175,688**	504,949	31,199	3,016,832	406,131
GSM1932400	E14_MS61T67	PostE_EPI	242	E14	around embryonic disk	141014ICSIday14A	male	GSE74767	6,640,905	**2,492,993**	1,224,128	50,531	2,244,773	628,480
GSM1932393	E14_MS13T76	PostE_EPI	243	E14	around embryonic disk	141014ICSIday14A	male	GSE74767	5,173,819	**2,230,982**	782,925	27,513	1,625,416	506,983
GSM1932398	E14_MS61T64	PostE_EPI	244	E14	around embryonic disk	141014ICSIday14A	male	GSE74767	4,674,366	**2,172,897**	676,874	16,119	1,325,248	483,228
GSM1932397	E14_MS61T63	PostE_EPI	245	E14	around embryonic disk	141014ICSIday14A	male	GSE74767	4,841,732	**2,501,774**	778,259	14,980	1,034,844	511,875
GSM1932406	E14_MS61T73	PostE_EPI	246	E14	around embryonic disk	141014ICSIday14A	male	GSE74767	7,329,080	**3,760,116**	1,056,217	29,739	1,749,026	733,982
GSM1932395	E14_MS61T60	PostE_EPI	247	E14	around embryonic disk	141014ICSIday14A	male	GSE74767	5,131,260	**2,793,393**	805,815	16,180	1,003,958	511,914
GSM1932403	E14_MS61T70	PostE_EPI	248	E14	around embryonic disk	141014ICSIday14A	male	GSE74767	5,648,592	**2,933,835**	805,367	20,691	1,357,164	531,535
GSM1932399	E14_MS61T66	PostE_EPI	249	E14	around embryonic disk	141014ICSIday14A	male	GSE74767	4,160,408	**1,765,840**	627,387	17,503	1,332,085	417,593
GSM1932405	E14_MS61T72	PostE_EPI	250	E14	around embryonic disk	141014ICSIday14A	male	GSE74767	4,732,250	**2,002,833**	608,464	17,101	1,695,533	408,319
GSM1932392	E14_MS13T74	PostE_EPI	251	E14	around embryonic disk	141014ICSIday14A	male	GSE74767	5,908,852	**2,812,008**	888,540	31,119	1,520,633	656,552
GSM1932396	E14_MS61T61	PostE_EPI	252	E14	around embryonic disk	141014ICSIday14A	male	GSE74767	4,009,583	**2,025,315**	639,702	20,422	958,211	365,933
GSM1932402	E14_MS61T69	PostE_EPI	253	E14	around embryonic disk	141014ICSIday14A	male	GSE74767	5,224,800	**2,543,266**	833,951	26,693	1,296,474	524,416
GSM1932401	E14_MS61T68	PostE_EPI	254	E14	around embryonic disk	141014ICSIday14A	male	GSE74767	5,762,852	**1,890,605**	730,323	26,877	2,584,245	530,802
GSM1932394	E14_MS13T78	PostE_EPI	255	E14	around embryonic disk	141014ICSIday14A	male	GSE74767	7,576,817	**2,359,981**	962,007	22,321	3,421,115	811,393
GSM1932404	E14_MS61T71	PostE_EPI	256	E14	around embryonic disk	141014ICSIday14A	male	GSE74767	5,875,217	**1,932,537**	711,004	28,833	2,764,747	438,096
GSM1932213	E13_MS61T45	Gast1	257	E13	around embryonic disk	140812ICSId13A	male	GSE74767	6,260,409	**2,796,039**	887,162	68,989	1,968,511	539,708
GSM1932214	E13_MS61T54	Gast1	258	E13	around embryonic disk	140812ICSId13A	male	GSE74767	4,067,745	**1,735,228**	591,561	42,163	1,371,322	327,471
GSM1932222	E14_MS13T87	Gast1	259	E14	around embryonic disk	141014ICSIday14A	male	GSE74767	5,590,610	**1,321,734**	434,019	28,733	3,389,510	416,614
GSM1932218	E14_MS13T80	Gast1	260	E14	around embryonic disk	141014ICSIday14A	male	GSE74767	6,766,479	**2,020,602**	724,000	36,047	3,386,343	599,487
GSM1932224	E14_MS61T65	Gast1	261	E14	around embryonic disk	141014ICSIday14A	male	GSE74767	4,349,151	**1,584,381**	645,839	15,429	1,695,124	408,378
GSM1932215	E14_MS13T75	Gast1	262	E14	around embryonic disk	141014ICSIday14A	male	GSE74767	5,166,036	**2,176,984**	765,430	23,296	1,636,849	563,477
GSM1932223	E14_MS13T90	Gast1	263	E14	around embryonic disk	141014ICSIday14A	male	GSE74767	5,972,781	**2,701,180**	735,771	24,314	1,894,707	616,809
GSM1932359	E13_MS10T50	Gast1	264	E13	around embryonic disk	140812ICSId13A	male	GSE74767	4,724,340	**1,712,771**	629,937	24,881	1,852,043	504,708
GSM1932219	E14_MS13T82	Gast1	265	E14	around embryonic disk	141014ICSIday14A	male	GSE74767	6,554,677	**2,366,708**	943,723	25,893	2,497,611	720,742
GSM1932221	E14_MS13T85	Gast1	266	E14	around embryonic disk	141014ICSIday14A	male	GSE74767	5,839,819	**2,117,626**	543,131	18,100	2,628,398	532,564
GSM1932217	E14_MS13T79	Gast1	267	E14	around embryonic disk	141014ICSIday14A	male	GSE74767	5,852,908	**2,454,754**	865,336	11,660	1,820,675	700,483
GSM1932353	E13_MS10T35	Gast1	268	E13	around embryonic disk	140812ICSId13A	male	GSE74767	4,200,696	**1,636,155**	615,928	19,349	1,458,497	470,767
GSM1932363	E13_MS10T55	Gast1	269	E13	around embryonic disk	140812ICSId13A	male	GSE74767	3,697,179	**1,663,284**	575,593	15,983	1,057,883	384,436
GSM1932216	E14_MS13T77	Gast1	270	E14	around embryonic disk	141014ICSIday14A	male	GSE74767	5,432,765	**1,961,603**	579,443	12,731	2,364,402	514,586
GSM1932220	E14_MS13T83	Gast1	271	E14	around embryonic disk	141014ICSIday14A	male	GSE74767	7,148,276	**3,241,380**	1,036,210	22,005	2,031,849	816,832
GSM1932419	E16_MS08T77	PostL_EPI	272	E16	around embryonic disk	140401ICSIday16	male	GSE74767	3,017,911	**1,284,591**	453,790	23,777	959,105	296,648
GSM1932420	E16_MS09T21	PostL_EPI	273	E16	around embryonic disk	140401ICSIday16	male	GSE74767	6,972,044	**2,169,463**	815,086	49,484	3,265,932	672,079
GSM1932226	E16_MS08T58	PostL_EPI	274	E16	around embryonic disk	140401ICSIday16	male	GSE74767	2,742,497	**1,454,138**	412,393	21,077	586,321	268,568
GSM1932415	E16_MS08T69	PostL_EPI	275	E16	around embryonic disk	140401ICSIday16	male	GSE74767	3,576,622	**1,175,118**	384,620	19,214	1,621,421	376,249
GSM1932225	E16_MS08T57	PostL_EPI	276	E16	around embryonic disk	140401ICSIday16	male	GSE74767	3,309,054	**1,324,410**	535,931	20,825	1,078,643	349,245
GSM1932227	E16_MS08T59	PostL_EPI	277	E16	around embryonic disk	140401ICSIday16	male	GSE74767	3,421,176	**1,130,905**	322,322	13,764	1,634,648	319,537
GSM1932228	E16_MS09T27	PostL_EPI	278	E16	around embryonic disk	140401ICSIday16	male	GSE74767	6,987,897	**3,190,969**	857,587	34,323	2,236,018	669,000
GSM1932417	E16_MS08T74	PostL_EPI	279	E16	around embryonic disk	140401ICSIday16	male	GSE74767	3,271,328	**1,122,744**	339,100	23,159	1,470,549	315,776
GSM1932414	E16_MS08T68	PostL_EPI	280	E16	around embryonic disk	140401ICSIday16	male	GSE74767	3,155,768	**1,297,779**	376,185	19,005	1,134,414	328,385
GSM1932408	E16_MS08T61	PostL_EPI	281	E16	around embryonic disk	140401ICSIday16	male	GSE74767	3,521,447	**1,089,438**	306,755	14,118	1,831,976	279,160
GSM1932411	E16_MS08T64	PostL_EPI	282	E16	around embryonic disk	140401ICSIday16	male	GSE74767	4,119,502	**1,491,255**	377,891	19,562	1,811,996	418,798
GSM1932423	E16_MS09T41	PostL_EPI	283	E16	around embryonic disk	140401ICSIday16	male	GSE74767	4,835,205	**2,365,878**	675,277	22,820	1,295,202	476,028
GSM1932409	E16_MS08T62	PostL_EPI	284	E16	around embryonic disk	140401ICSIday16	male	GSE74767	4,383,791	**1,783,961**	586,778	16,350	1,549,661	447,041
GSM1932412	E16_MS08T65	PostL_EPI	285	E16	around embryonic disk	140401ICSIday16	male	GSE74767	4,660,737	**2,301,288**	754,680	20,951	1,098,067	485,751
GSM1932416	E16_MS08T71	PostL_EPI	286	E16	around embryonic disk	140401ICSIday16	male	GSE74767	3,872,999	**1,407,729**	452,015	19,419	1,610,000	383,836
GSM1932421	E16_MS09T28	PostL_EPI	287	E16	around embryonic disk	140401ICSIday16	male	GSE74767	5,712,754	**1,553,723**	520,191	18,892	3,074,141	545,807
GSM1932410	E16_MS08T63	PostL_EPI	288	E16	around embryonic disk	140401ICSIday16	male	GSE74767	4,274,353	**2,069,039**	621,291	25,384	1,101,209	457,430
GSM1932413	E16_MS08T66	PostL_EPI	289	E16	around embryonic disk	140401ICSIday16	male	GSE74767	3,962,474	**1,750,334**	500,422	19,317	1,287,892	404,509
GSM1932422	E16_MS09T36	PostL_EPI	290	E16	around embryonic disk	140401ICSIday16	male	GSE74767	6,134,019	**2,550,707**	884,771	57,696	2,083,739	557,106
GSM1932407	E16_MS08T60	PostL_EPI	291	E16	around embryonic disk	140401ICSIday16	male	GSE74767	4,836,244	**1,823,887**	499,567	41,495	2,026,415	444,880
GSM1932418	E16_MS08T75	PostL_EPI	292	E16	around embryonic disk	140401ICSIday16	male	GSE74767	2,493,261	**1,156,986**	419,735	13,778	656,578	246,184
GSM1932432	E17_MS10T84	PostL_EPI	293	E17	around embryonic disk	140722ICSIday17A	male	GSE74767	4,023,290	**1,750,881**	632,621	10,883	1,199,525	429,380
GSM1932442	E17_MS11T49	PostL_EPI	294	E17	around embryonic disk	140722ICSIday17A	male	GSE74767	6,332,238	**2,812,972**	955,759	16,352	1,922,568	624,587
GSM1932443	E17_MS11T78	PostL_EPI	295	E17	around embryonic disk	140722ICSIday17A	male	GSE74767	7,752,671	**3,068,219**	1,036,405	29,444	2,838,819	779,784
GSM1932447	E17_MS11T84	PostL_EPI	296	E17	around embryonic disk	140722ICSIday17A	male	GSE74767	5,943,922	**2,536,880**	959,643	19,565	1,758,741	669,093
GSM1932424	E17_MS10T66	PostL_EPI	297	E17	around embryonic disk	140722ICSIday17A	male	GSE74767	4,123,632	**1,717,130**	616,864	5,734	1,309,485	474,419
GSM1932229	E17_MS11T77	PostL_EPI	298	E17	around embryonic disk	140722ICSIday17A	male	GSE74767	5,779,221	**2,515,040**	978,086	15,745	1,712,081	558,269
GSM1932230	E17_MS11T82	PostL_EPI	299	E17	around embryonic disk	140722ICSIday17A	male	GSE74767	5,760,027	**2,498,382**	843,698	11,423	1,818,088	588,436
GSM1932429	E17_MS10T77	PostL_EPI	300	E17	around embryonic disk	140722ICSIday17A	male	GSE74767	4,023,569	**2,003,710**	657,280	9,679	951,445	401,455
GSM1932431	E17_MS10T82	PostL_EPI	301	E17	around embryonic disk	140722ICSIday17A	male	GSE74767	4,761,433	**1,853,833**	580,120	8,003	1,876,707	442,770
GSM1932454	E17_MS61T42	PostL_EPI	302	E17	around embryonic disk	140722ICSIday17A	male	GSE74767	5,839,848	**2,869,059**	854,783	11,588	1,431,495	672,923
GSM1932455	E17_MS61T43	PostL_EPI	303	E17	around embryonic disk	140722ICSIday17A	male	GSE74767	4,802,883	**1,528,440**	569,801	9,119	2,211,970	483,553
GSM1932452	E17_MS61T40	PostL_EPI	304	E17	around embryonic disk	140722ICSIday17A	male	GSE74767	7,816,409	**4,013,305**	1,614,145	16,708	1,365,709	806,542
GSM1932426	E17_MS10T71	PostL_EPI	305	E17	around embryonic disk	140722ICSIday17A	male	GSE74767	3,960,544	**1,680,169**	536,347	6,610	1,351,425	385,993
GSM1932435	E17_MS10T87	PostL_EPI	306	E17	around embryonic disk	140722ICSIday17A	male	GSE74767	4,062,660	**1,878,167**	634,085	6,605	1,143,165	400,638
GSM1932440	E17_MS10T94	PostL_EPI	307	E17	around embryonic disk	140722ICSIday17A	male	GSE74767	3,835,690	**1,868,758**	598,493	7,673	966,778	393,988
GSM1932439	E17_MS10T93	PostL_EPI	308	E17	around embryonic disk	140722ICSIday17A	male	GSE74767	4,368,840	**1,868,871**	703,941	6,928	1,361,031	428,069
GSM1932441	E17_MS11T46	PostL_EPI	309	E17	around embryonic disk	140722ICSIday17A	male	GSE74767	4,819,748	**2,325,097**	687,338	8,723	1,351,647	446,943
GSM1932425	E17_MS10T68	PostL_EPI	310	E17	around embryonic disk	140722ICSIday17A	male	GSE74767	4,314,460	**1,988,346**	699,701	7,463	1,168,035	450,915
GSM1932445	E17_MS11T80	PostL_EPI	311	E17	around embryonic disk	140722ICSIday17A	male	GSE74767	6,360,692	**2,770,899**	1,028,820	10,546	1,923,227	627,200
GSM1932438	E17_MS10T92	PostL_EPI	312	E17	around embryonic disk	140722ICSIday17A	male	GSE74767	4,056,315	**1,738,857**	658,106	5,791	1,207,803	445,758
GSM1932450	E17_MS61T38	PostL_EPI	313	E17	around embryonic disk	140722ICSIday17A	male	GSE74767	6,802,869	**3,662,405**	1,232,000	14,467	1,193,026	700,971
GSM1932451	E17_MS61T39	PostL_EPI	314	E17	around embryonic disk	140722ICSIday17A	male	GSE74767	4,388,044	**2,345,632**	780,819	9,864	854,664	397,065
GSM1932453	E17_MS61T41	PostL_EPI	315	E17	around embryonic disk	140722ICSIday17A	male	GSE74767	6,199,367	**2,871,477**	1,024,328	6,945	1,637,822	658,795
GSM1932456	E17_MS61T62	PostL_EPI	316	E17	around embryonic disk	140722ICSIday17A	male	GSE74767	5,438,048	**2,796,011**	1,041,016	9,925	987,067	604,029
GSM1932437	E17_MS10T91	PostL_EPI	317	E17	around embryonic disk	140722ICSIday17A	male	GSE74767	3,281,102	**1,523,138**	547,329	4,038	881,090	325,507
GSM1932446	E17_MS11T81	PostL_EPI	318	E17	around embryonic disk	140722ICSIday17A	male	GSE74767	7,434,085	**2,926,883**	857,048	19,107	2,891,178	739,869
GSM1932436	E17_MS10T88	PostL_EPI	319	E17	around embryonic disk	140722ICSIday17A	male	GSE74767	9,291,965	**3,534,721**	1,299,099	11,996	3,438,825	1,007,324
GSM1932444	E17_MS11T79	PostL_EPI	320	E17	around embryonic disk	140722ICSIday17A	male	GSE74767	7,011,810	**3,495,850**	1,077,672	7,063	1,692,890	738,335
GSM1932428	E17_MS10T73	PostL_EPI	321	E17	around embryonic disk	140722ICSIday17A	male	GSE74767	4,991,604	**2,405,558**	754,428	3,101	1,275,170	553,347
GSM1932448	E17_MS13T95	PostL_EPI	322	E17	around embryonic disk	140722ICSIday17A	male	GSE74767	2,818,753	**1,484,505**	522,896	1,438	501,125	308,789
GSM1932434	E17_MS10T86	PostL_EPI	323	E17	around embryonic disk	140722ICSIday17A	male	GSE74767	3,919,946	**1,161,278**	775,011	5,914	1,502,712	475,031
GSM1932427	E17_MS10T72	PostL_EPI	324	E17	around embryonic disk	140722ICSIday17A	male	GSE74767	4,853,357	**2,041,471**	665,068	10,098	1,640,388	496,332
GSM1932433	E17_MS10T85	PostL_EPI	325	E17	around embryonic disk	140722ICSIday17A	male	GSE74767	4,018,000	**1,408,426**	650,193	7,385	1,531,369	420,627
GSM1932430	E17_MS10T81	PostL_EPI	326	E17	around embryonic disk	140722ICSIday17A	male	GSE74767	10,177,704	**4,585,826**	1,764,589	19,875	2,650,876	1,156,538
GSM1932449	E17_MS61T37	PostL_EPI	327	E17	around embryonic disk	140722ICSIday17A	male	GSE74767	5,771,399	**2,997,376**	1,103,821	10,191	1,047,149	612,862
GSM1932150	C6FF_MS11T85	cyESC	328	p35	on feeder	CMK6	male	GSE74767	5,685,879	**1,394,685**	762,044	72,737	2,997,581	458,832
GSM1932157	C6FF_MS11T94	cyESC	329	p35	on feeder	CMK6	male	GSE74767	5,399,985	**1,666,197**	750,437	58,148	2,474,841	450,362
GSM1932151	C6FF_MS11T86	cyESC	330	p35	on feeder	CMK6	male	GSE74767	4,718,245	**1,290,189**	983,718	30,524	1,965,411	448,403
GSM1932154	C6FF_MS11T89	cyESC	331	p35	on feeder	CMK6	male	GSE74767	7,617,392	**2,282,589**	1,209,734	52,433	3,221,062	851,574
GSM1932155	C6FF_MS11T91	cyESC	332	p35	on feeder	CMK6	male	GSE74767	6,165,768	**2,344,540**	871,538	45,552	2,363,629	540,509
GSM1932153	C6FF_MS11T88	cyESC	333	p35	on feeder	CMK6	male	GSE74767	6,568,211	**2,610,226**	1,110,630	46,406	2,161,056	639,893
GSM1932152	C6FF_MS11T87	cyESC	334	p35	on feeder	CMK6	male	GSE74767	9,385,186	**3,951,387**	1,527,775	55,172	2,993,424	857,428
GSM1932156	C6FF_MS11T92	cyESC	335	p35	on feeder	CMK6	male	GSE74767	7,049,544	**2,571,496**	955,412	41,141	2,813,948	667,547
GSM1932158	C6FF_MS11T95	cyESC	336	p35	on feeder	CMK6	male	GSE74767	5,765,027	**2,174,233**	820,136	39,835	2,258,408	472,415
GSM1932166	C6oF_MS05T60	cyESC	337	p32	on feeder	CMK6	male	GSE74767	5,602,944	**2,038,658**	853,198	81,768	1,999,860	629,460
GSM1932170	C6oF_MS05T65	cyESC	338	p32	on feeder	CMK6	male	GSE74767	4,338,016	**1,872,461**	732,407	73,732	1,150,358	509,058
GSM1932161	C6oF_MS05T55	cyESC	339	p32	on feeder	CMK6	male	GSE74767	4,573,153	**1,803,673**	808,437	74,413	1,357,596	529,034
GSM1932164	C6oF_MS05T58	cyESC	340	p33	on feeder	CMK6	male	GSE74767	5,260,014	**2,402,288**	1,088,409	57,867	1,095,462	615,988
GSM1932160	C6oF_MS05T54	cyESC	341	p32	on feeder	CMK6	male	GSE74767	4,252,513	**1,868,236**	747,226	58,557	1,097,275	481,219
GSM1932171	C6oF_MS05T66	cyESC	342	p32	on feeder	CMK6	male	GSE74767	6,482,149	**2,543,430**	1,177,311	77,141	1,862,423	821,844
GSM1932165	C6oF_MS05T59	cyESC	343	p33	on feeder	CMK6	male	GSE74767	4,931,267	**1,972,305**	790,650	47,680	1,459,006	661,626
GSM1932163	C6oF_MS05T57	cyESC	344	p33	on feeder	CMK6	male	GSE74767	5,926,478	**2,280,339**	855,457	56,093	2,067,006	667,583
GSM1932159	C6oF_MS05T53	cyESC	345	p32	on feeder	CMK6	male	GSE74767	4,946,669	**1,916,811**	1,362,942	50,225	1,011,756	604,935
GSM1932162	C6oF_MS05T56	cyESC	346	p33	on feeder	CMK6	male	GSE74767	5,493,273	**2,518,346**	932,134	54,525	1,281,251	707,017
GSM1932168	C6oF_MS05T63	cyESC	347	p32	on feeder	CMK6	male	GSE74767	7,969,936	**3,146,701**	1,089,650	139,992	2,679,399	914,194
GSM1932172	C6oF_MS05T67	cyESC	348	p32	on feeder	CMK6	male	GSE74767	7,307,147	**3,357,337**	984,953	128,640	2,021,391	814,826
GSM1932167	C6oF_MS05T61	cyESC	349	p32	on feeder	CMK6	male	GSE74767	6,181,340	**2,867,046**	957,189	97,759	1,639,481	619,865
GSM1932169	C6oF_MS05T64	cyESC	350	p32	on feeder	CMK6	male	GSE74767	6,002,302	**2,547,962**	856,777	83,920	1,801,952	711,691
GSM1932180	CMK9_MS05T75	cyESC	351	p30	on feeder	CMK9	female	GSE74767	7,016,731	**2,694,784**	1,161,606	120,177	2,233,176	806,988
GSM1932174	CMK9_MS05T69	cyESC	352	p25	on feeder	CMK9	female	GSE74767	5,994,987	**2,640,013**	774,385	85,814	1,822,376	672,399
GSM1932177	CMK9_MS05T72	cyESC	353	p30	on feeder	CMK9	female	GSE74767	5,917,510	**2,083,645**	785,929	89,618	2,321,327	636,991
GSM1932178	CMK9_MS05T73	cyESC	354	p30	on feeder	CMK9	female	GSE74767	6,484,221	**2,576,534**	1,059,246	85,767	2,016,955	745,719
GSM1932179	CMK9_MS05T74	cyESC	355	p30	on feeder	CMK9	female	GSE74767	6,377,212	**1,744,318**	712,750	60,254	3,203,996	655,894
GSM1932173	CMK9_MS05T68	cyESC	356	p25	on feeder	CMK9	female	GSE74767	6,015,452	**2,546,174**	719,918	59,132	2,005,387	684,841
GSM1932175	CMK9_MS05T70	cyESC	357	p25	on feeder	CMK9	female	GSE74767	6,520,471	**3,392,703**	915,233	48,455	1,379,355	784,725
GSM1932176	CMK9_MS05T71	cyESC	358	p30	on feeder	CMK9	female	GSE74767	6,099,778	**2,032,247**	1,008,912	39,444	2,313,087	706,088
GSM1932243	E17_MS11T48	Gast2a	359	E17	around embryonic disk	140722ICSIday17A	male	GSE74767	5,806,294	**2,678,662**	846,001	12,627	1,636,045	632,959
GSM1932238	E17_MS10T70	Gast2a	360	E17	around embryonic disk	140722ICSIday17A	male	GSE74767	2,294,680	**895,882**	307,165	8,389	853,773	229,471
GSM1932239	E17_MS10T75	Gast2a	361	E17	around embryonic disk	140722ICSIday17A	male	GSE74767	3,944,616	**1,858,387**	640,854	3,601	1,040,613	401,161
GSM1932233	E16_MS09T30	Gast2a	362	E16	around embryonic disk	140401ICSIday16	male	GSE74767	8,574,550	**2,828,497**	803,937	24,489	4,195,868	721,759
GSM1932241	E17_MS10T89	Gast2a	363	E17	around embryonic disk	140722ICSIday17A	male	GSE74767	3,919,592	**1,754,548**	673,529	4,666	1,064,154	422,695
GSM1978223	E16_MS09T61	Gast2a	364	E16	posterior third of embryonic disk	140708ICSIday16	female	GSE76267	4,049,199	**2,282,575**	853,571	267	560,632	352,154
GSM1932235	E17_MS10T65	Gast2a	365	E17	around embryonic disk	140722ICSIday17A	male	GSE74767	4,440,931	**2,268,581**	538,336	6,382	1,210,161	417,471
GSM1932237	E17_MS10T69	Gast2a	366	E17	around embryonic disk	140722ICSIday17A	male	GSE74767	4,433,787	**1,199,634**	448,072	3,640	2,367,667	414,774
GSM1932231	E16_MS08T72	Gast2a	367	E16	around embryonic disk	140401ICSIday16	male	GSE74767	3,659,156	**627,889**	165,971	9,682	2,619,601	236,013
GSM1978241	E17_MS11T51	Gast2a	368	E17	around embryonic disk	140722ICSIday17A	male	GSE76267	5,841,383	**2,885,799**	986,986	10,887	1,364,571	593,140
GSM1932232	E16_MS08T73	Gast2a	369	E16	around embryonic disk	140401ICSIday16	male	GSE74767	3,337,171	**961,445**	293,657	17,746	1,748,821	315,502
GSM1932240	E17_MS10T76	Gast2a	370	E17	around embryonic disk	140722ICSIday17A	male	GSE74767	3,057,128	**1,377,378**	463,617	8,849	930,996	276,288
GSM1932242	E17_MS11T47	Gast2a	371	E17	around embryonic disk	140722ICSIday17A	male	GSE74767	6,081,119	**3,015,809**	909,886	12,009	1,543,543	599,872
GSM1932234	E17_MS10T64	Gast2a	372	E17	around embryonic disk	140722ICSIday17A	male	GSE74767	4,683,382	**1,799,438**	610,713	9,817	1,755,512	507,902
GSM1932236	E17_MS10T67	Gast2a	373	E17	around embryonic disk	140722ICSIday17A	male	GSE74767	3,571,880	**1,451,245**	431,905	8,664	1,376,458	303,608
GSM1932252	E17_MS10T95	Gast2b	374	E17	around embryonic disk	140722ICSIday17A	male	GSE74767	3,682,906	**790,702**	251,159	3,396	2,378,270	259,379
GSM1932255	E17_MS13T94	Gast2b	375	E17	around embryonic disk	140722ICSIday17A	male	GSE74767	5,297,975	**2,463,188**	942,433	8,642	1,216,573	667,139
GSM1932245	E16_MS08T76	Gast2b	376	E16	around embryonic disk	140401ICSIday16	male	GSE74767	2,299,055	**604,446**	193,625	10,199	1,272,468	218,317
GSM1932246	E16_MS09T22	Gast2b	377	E16	around embryonic disk	140401ICSIday16	male	GSE74767	4,897,138	**1,742,463**	461,643	31,755	2,208,400	452,877
GSM1932256	E17_MS13T96	Gast2b	378	E17	around embryonic disk	140722ICSIday17A	male	GSE74767	3,365,476	**1,423,386**	524,005	10,309	1,060,600	347,176
GSM1932248	E16_MS09T25	Gast2b	379	E16	around embryonic disk	140401ICSIday16	male	GSE74767	5,437,975	**1,113,448**	370,742	22,315	3,496,380	435,090
GSM1932251	E17_MS10T83	Gast2b	380	E17	around embryonic disk	140722ICSIday17A	male	GSE74767	4,855,016	**1,984,269**	658,425	9,846	1,706,078	496,398
GSM1932250	E17_MS10T78	Gast2b	381	E17	around embryonic disk	140722ICSIday17A	male	GSE74767	4,682,344	**2,164,705**	656,983	7,979	1,341,594	511,083
GSM1932244	E16_MS08T67	Gast2b	382	E16	around embryonic disk	140401ICSIday16	male	GSE74767	4,073,298	**1,422,526**	361,201	14,969	1,827,696	446,906
GSM1932247	E16_MS09T24	Gast2b	383	E16	around embryonic disk	140401ICSIday16	male	GSE74767	5,581,486	**2,005,020**	522,470	22,318	2,546,170	485,508
GSM1932249	E16_MS09T33	Gast2b	384	E16	around embryonic disk	140401ICSIday16	male	GSE74767	8,922,904	**2,781,866**	861,468	46,039	4,401,670	831,861
GSM1932253	E17_MS10T96	Gast2b	385	E17	around embryonic disk	140722ICSIday17A	male	GSE74767	1,652,495	**753,658**	308,308	3,416	411,239	175,874
GSM1932254	E17_MS11T83	Gast2b	386	E17	around embryonic disk	140722ICSIday17A	male	GSE74767	6,352,155	**2,446,879**	959,800	13,730	2,301,042	630,704
GSM1978213	E20_MS08T83	Gast2b	387	E20	around body stalk	140401ICSIday20	male	GSE76267	2,533,340	**1,222,210**	462,118	17,427	546,024	285,561
GSM1978211	E20_MS08T81	Gast2b	388	E20	around body stalk	140401ICSIday20	male	GSE76267	3,415,819	**1,614,001**	579,720	19,755	844,059	358,284
GSM1978209	E20_MS08T78	Gast2b	389	E20	around body stalk	140401ICSIday20	male	GSE76267	2,877,068	**1,491,499**	558,264	2,960	515,863	308,482
GSM1978212	E20_MS08T82	Gast2b	390	E20	around body stalk	140401ICSIday20	male	GSE76267	2,913,013	**1,373,265**	415,812	11,837	817,774	294,325
GSM1978210	E20_MS08T79	Gast2b	391	E20	around body stalk	140401ICSIday20	male	GSE76267	3,711,400	**1,396,069**	459,471	35,709	1,472,680	347,471
GSM1978214	E20_MS08T84	Gast2b	392	E20	around body stalk	140401ICSIday20	male	GSE76267	3,830,874	**1,522,648**	522,212	32,553	1,362,770	390,691
GSM1978215	E20_MS08T85	Gast2b	393	E20	around body stalk	140401ICSIday20	male	GSE76267	3,347,144	**1,107,501**	455,724	32,267	1,459,582	292,070
GSM1932570	E17_MS13T91	VEYE	394	E17	around embryonic disk	140722ICSIday17A	male	GSE74767	5,370,495	**2,364,228**	760,318	27,171	1,646,584	572,194
GSM1932569	E16_MS09T31	VEYE	395	E16	around embryonic disk	140401ICSIday16	male	GSE74767	6,719,326	**1,904,855**	497,391	61,908	3,651,103	604,069
GSM1932567	E14_MS09T18	VEYE	396	E14	around embryonic disk	140401ICSIday14	male	GSE74767	8,900,878	**2,724,546**	1,025,611	81,392	4,094,701	974,628
GSM1932566	E13_MS13T65	VEYE	397	E13	around embryonic disk	140812ICSId13A	male	GSE74767	6,550,968	**2,745,730**	866,410	74,838	2,183,023	680,967
GSM1932568	E16_MS09T29	VEYE	398	E16	around embryonic disk	140401ICSIday16	male	GSE74767	4,699,044	**1,326,601**	497,129	27,374	2,413,441	434,499
GSM1932196	E13_MS61T51	EXMC	399	E13	around embryonic disk	140812ICSId13A	male	GSE74767	2,380,449	**649,422**	505,261	35,654	935,396	254,716
GSM1932193	E13_MS61T44	EXMC	400	E13	around embryonic disk	140812ICSId13A	male	GSE74767	4,992,419	**1,777,702**	1,014,748	61,755	1,511,700	626,514
GSM1932192	E13_MS13T73	EXMC	401	E13	around embryonic disk	140812ICSId13A	male	GSE74767	4,968,605	**1,565,778**	570,780	31,702	2,089,133	711,212
GSM1932194	E13_MS61T49	EXMC	402	E13	around embryonic disk	140812ICSId13A	male	GSE74767	4,514,622	**1,726,413**	587,602	39,365	1,791,584	369,658
GSM1932188	E13_MS13T69	EXMC	403	E13	around embryonic disk	140812ICSId13A	male	GSE74767	7,418,081	**3,187,534**	981,406	59,464	2,389,629	800,048
GSM1932189	E13_MS13T70	EXMC	404	E13	around embryonic disk	140812ICSId13A	male	GSE74767	5,351,896	**2,036,025**	612,586	41,610	2,112,910	548,765
GSM1932187	E13_MS13T68	EXMC	405	E13	around embryonic disk	140812ICSId13A	male	GSE74767	4,728,036	**1,696,836**	539,997	37,436	1,972,157	481,610
GSM1932195	E13_MS61T50	EXMC	406	E13	around embryonic disk	140812ICSId13A	male	GSE74767	5,113,147	**2,568,357**	856,786	42,108	1,124,353	521,543
GSM1932183	E13_MS10T48	EXMC	407	E13	around embryonic disk	140812ICSId13A	male	GSE74767	5,003,608	**1,667,614**	505,878	28,894	2,299,225	501,997
GSM1932186	E13_MS13T67	EXMC	408	E13	around embryonic disk	140812ICSId13A	male	GSE74767	7,259,032	**2,935,365**	947,510	53,442	2,528,829	793,886
GSM1932191	E13_MS13T72	EXMC	409	E13	around embryonic disk	140812ICSId13A	male	GSE74767	3,647,420	**1,351,719**	436,631	23,525	1,477,061	358,484
GSM1932182	E13_MS10T46	EXMC	410	E13	around embryonic disk	140812ICSId13A	male	GSE74767	3,462,825	**1,558,079**	482,451	22,280	1,042,687	357,328
GSM1932181	E13_MS10T45	EXMC	411	E13	around embryonic disk	140812ICSId13A	male	GSE74767	4,215,974	**1,682,418**	602,849	17,471	1,430,047	483,189
GSM1932184	E13_MS10T49	EXMC	412	E13	around embryonic disk	140812ICSId13A	male	GSE74767	3,890,228	**1,699,838**	480,106	19,696	1,319,844	370,744
GSM1932185	E13_MS13T66	EXMC	413	E13	around embryonic disk	140812ICSId13A	male	GSE74767	6,730,740	**2,073,784**	639,340	32,378	3,356,625	628,613
GSM1932190	E13_MS13T71	EXMC	414	E13	around embryonic disk	140812ICSId13A	male	GSE74767	5,010,347	**1,782,709**	514,103	30,293	2,213,392	469,850
GSM1932201	E14_MS09T12	EXMC	415	E14	around embryonic disk	140401ICSIday14	male	GSE74767	4,499,258	**1,396,689**	673,212	47,127	1,909,433	472,797
GSM1932198	E14_MS09T05	EXMC	416	E14	around embryonic disk	140401ICSIday14	male	GSE74767	4,712,170	**1,972,492**	726,892	32,778	1,421,622	558,386
GSM1932202	E14_MS09T13	EXMC	417	E14	around embryonic disk	140401ICSIday14	male	GSE74767	4,887,552	**1,994,152**	611,182	30,203	1,732,975	519,040
GSM1932197	E14_MS09T03	EXMC	418	E14	around embryonic disk	140401ICSIday14	male	GSE74767	6,179,641	**2,674,674**	922,508	38,436	1,809,175	734,848
GSM1932199	E14_MS09T08	EXMC	419	E14	around embryonic disk	140401ICSIday14	male	GSE74767	6,284,532	**2,799,760**	1,035,924	38,507	1,673,776	736,565
GSM1932200	E14_MS09T09	EXMC	420	E14	around embryonic disk	140401ICSIday14	male	GSE74767	5,235,294	**1,435,777**	1,072,389	30,040	1,885,009	812,079
GSM1932212	E16_MS09T40	EXMC	421	E16	around embryonic disk	140401ICSIday16	male	GSE74767	6,122,799	**2,420,950**	678,658	55,194	2,425,850	542,147
GSM1932208	E14_MS09T20	EXMC	422	E14	around embryonic disk	140401ICSIday14	male	GSE74767	5,951,877	**1,828,462**	1,514,995	71,181	1,703,653	833,586
GSM1932211	E16_MS09T35	EXMC	423	E16	around embryonic disk	140401ICSIday16	male	GSE74767	5,783,250	**1,645,154**	595,237	37,661	3,030,070	475,128
GSM1932210	E14_MS13T89	EXMC	424	E14	around embryonic disk	141014ICSIday14A	male	GSE74767	5,107,578	**1,751,425**	561,929	30,353	2,284,863	479,008
GSM1932203	E14_MS09T14	EXMC	425	E14	around embryonic disk	140401ICSIday14	male	GSE74767	8,424,537	**3,525,377**	1,339,289	64,959	2,522,369	972,543
GSM1932205	E14_MS09T16	EXMC	426	E14	around embryonic disk	140401ICSIday14	male	GSE74767	6,405,722	**2,397,545**	975,445	46,620	2,132,229	853,883
GSM1932207	E14_MS09T19	EXMC	427	E14	around embryonic disk	140401ICSIday14	male	GSE74767	4,994,805	**1,689,785**	873,867	25,957	1,794,598	610,598
GSM1932209	E14_MS13T88	EXMC	428	E14	around embryonic disk	141014ICSIday14A	male	GSE74767	5,374,005	**2,084,772**	649,894	20,540	2,102,412	516,387
GSM1932204	E14_MS09T15	EXMC	429	E14	around embryonic disk	140401ICSIday14	male	GSE74767	7,495,611	**3,087,547**	1,692,548	42,352	1,720,382	952,782
GSM1932206	E14_MS09T17	EXMC	430	E14	around embryonic disk	140401ICSIday14	male	GSE74767	6,623,690	**2,835,967**	1,161,044	43,281	1,855,882	727,516
GSM1978240	E17_MS11T50	ePGC	431	E17	around embryonic disk	140722ICSIday17A	male	GSE76267	6,484,754	**2,759,696**	1,025,123	32,728	2,042,132	625,075
GSM1978227	E20_MS09T80	ePGC	432	E20	around body stalk	140401ICSIday20	male	GSE76267	20,379,189	**2,768,207**	1,134,960	55,288	15,228,572	1,192,162
GSM1978242	E14_MS13T84	ePGC	433	E14	around embryonic disk	141014ICSIday14A	male	GSE76267	6,451,769	**2,371,638**	989,233	28,464	2,312,769	749,665
GSM1978229	E13_MS10T41	ePGC	434	E13	around embryonic disk	140812ICSId13A	male	GSE76267	6,195,292	**1,832,200**	745,359	53,311	2,891,781	672,641
GSM1978228	E13_MS10T38	ePGC	435	E13	around embryonic disk	140812ICSId13A	male	GSE76267	4,544,428	**1,348,416**	474,886	34,187	2,300,868	386,071
GSM1978230	E13_MS10T42	ePGC	436	E13	around embryonic disk	140812ICSId13A	male	GSE76267	5,397,010	**1,793,362**	669,596	34,896	2,292,925	606,231
GSM1978221	E16_MS09T59	ePGC	437	E16	posterior third of embryonic disk	140708ICSIday16	female	GSE76267	4,725,321	**1,976,399**	912,879	7,891	1,380,225	447,927
GSM1978219	E16_MS09T57	ePGC	438	E16	posterior third of embryonic disk	140708ICSIday16	female	GSE76267	6,592,996	**2,471,100**	1,046,508	10,372	2,502,009	563,007
GSM1978220	E16_MS09T58	ePGC	439	E16	posterior third of embryonic disk	140708ICSIday16	female	GSE76267	5,846,395	**2,445,922**	1,061,205	9,531	1,788,066	541,671
GSM1978216	E16_MS09T54	ePGC	440	E16	posterior third of embryonic disk	140708ICSIday16	female	GSE76267	6,440,845	**2,768,662**	1,250,856	11,287	1,828,087	581,953
GSM1978218	E16_MS09T56	ePGC	441	E16	posterior third of embryonic disk	140708ICSIday16	female	GSE76267	7,420,875	**3,629,404**	1,440,353	8,838	1,606,541	735,739
GSM1978224	E16_MS09T62	ePGC	442	E17	around body stalk	140701ICSIday17	female	GSE76267	6,593,092	**3,223,754**	1,231,392	11,764	1,493,770	632,412
GSM1978225	E16_MS09T63	ePGC	443	E17	around body stalk	140701ICSIday17	female	GSE76267	4,563,768	**2,197,235**	678,906	8,189	1,241,506	437,932
GSM1978226	E16_MS09T64	ePGC	444	E17	around body stalk	140701ICSIday17	female	GSE76267	4,933,267	**2,292,021**	797,954	8,037	1,357,713	477,542
GSM1978217	E16_MS09T55	ePGC	445	E16	posterior third of embryonic disk	140708ICSIday16	female	GSE76267	4,860,698	**2,001,514**	878,010	8,010	1,542,364	430,800
GSM1978222	E16_MS09T60	ePGC	446	E16	posterior third of embryonic disk	140708ICSIday16	female	GSE76267	5,728,320	**2,418,845**	1,280,759	10,686	1,480,333	537,697
GSM1978231	E36m_MS11T36	lPGC	447	E36	genital ridge	140708ICSId36#60	male	GSE76267	5,400,628	**2,564,184**	719,087	88,017	1,615,308	414,032
GSM1978233	E36m_MS11T39	lPGC	448	E36	genital ridge	140708ICSId36#60	male	GSE76267	4,484,534	**2,101,368**	659,813	53,644	1,351,214	318,495
GSM1978234	E36m_MS11T40	lPGC	449	E36	genital ridge	140708ICSId36#60	male	GSE76267	5,367,304	**2,674,202**	883,623	74,230	1,293,189	442,060
GSM1978232	E36m_MS11T37	lPGC	450	E36	genital ridge	140708ICSId36#60	male	GSE76267	4,398,119	**838,423**	233,628	24,041	3,044,209	257,818
GSM1978236	E36m_MS11T42	lPGC	451	E36	genital ridge	140708ICSId36#60	male	GSE76267	4,060,696	**835,082**	255,584	20,899	2,730,162	218,969
GSM1978235	E36m_MS11T41	lPGC	452	E36	genital ridge	140708ICSId36#60	male	GSE76267	6,465,443	**3,042,654**	1,113,433	59,106	1,693,668	556,582
GSM1643205	D51_MS04T45	lPGC	453	E51	genital ridge	130522ICSI-PGCday51	female	GSE67259	6,217,902	**1,987,429**	953,264	48,821	2,421,909	806,479
GSM1978237	E47m_MS11T43	lPGC	454	E47	genital ridge	130522ICSI-PGCday47	male	GSE76267	4,906,783	**1,142,966**	471,600	30,758	2,847,557	413,902
GSM1643192	D43_MS04T07	lPGC	455	E43	genital ridge	130522ICSI-PGCday43	female	GSE67259	5,257,575	**1,388,708**	600,162	59,621	2,738,634	470,450
GSM1643189	D43_MS04T03	lPGC	456	E43	genital ridge	130522ICSI-PGCday43	female	GSE67259	7,810,554	**2,442,060**	836,572	92,390	3,690,427	749,105
GSM1643193	D43_MS04T08	lPGC	457	E43	genital ridge	130522ICSI-PGCday43	female	GSE67259	5,315,584	**2,054,875**	818,982	50,805	1,852,925	537,997
GSM1643188	D43_MS04T02	lPGC	458	E43	genital ridge	130522ICSI-PGCday43	female	GSE67259	6,937,663	**2,311,985**	863,645	67,975	3,022,088	671,970
GSM1643190	D43_MS04T04	lPGC	459	E43	genital ridge	130522ICSI-PGCday43	female	GSE67259	7,382,718	**2,568,659**	1,245,873	62,589	2,833,619	671,978
GSM1643195	D50_MS04T13	lPGC	460	E50	genital ridge	130523ICSI-PGCAday50	female	GSE67259	5,032,528	**658,565**	286,527	57,956	3,612,581	416,899
GSM1643201	D51_MS04T12	lPGC	461	E51	genital ridge	130522ICSI-PGCday51	female	GSE67259	5,051,665	**1,272,937**	447,202	78,010	2,843,959	409,557
GSM1643200	D51_MS04T05	lPGC	462	E51	genital ridge	130522ICSI-PGCday51	female	GSE67259	5,158,521	**1,146,301**	429,323	76,297	3,065,474	441,126
GSM1643203	D51_MS04T32	lPGC	463	E51	genital ridge	130522ICSI-PGCday51	female	GSE67259	4,714,689	**1,255,199**	469,655	53,814	2,582,227	353,794
GSM1978238	E55m_MS11T44	lPGC	464	E55	genital ridge	130522ICSI-PGCAday55	male	GSE76267	4,192,986	**1,006,194**	281,403	49,950	2,546,091	309,348
GSM1643204	D51_MS04T44	lPGC	465	E51	genital ridge	130522ICSI-PGCday51	female	GSE67259	5,838,129	**1,445,421**	618,080	76,858	3,186,196	511,574
GSM1643202	D51_MS04T25	lPGC	466	E51	genital ridge	130522ICSI-PGCday51	female	GSE67259	4,819,220	**1,697,101**	740,520	60,610	1,831,995	488,994
GSM1643206	D51_MS04T51	lPGC	467	E51	genital ridge	130522ICSI-PGCday51	female	GSE67259	5,931,039	**2,780,764**	970,445	79,686	1,557,214	542,930
GSM1978239	E55m_MS11T45	lPGC	468	E55	genital ridge	130522ICSI-PGCBday55	male	GSE76267	5,041,062	**1,427,413**	450,922	49,535	2,744,972	368,220
GSM1643199	D50_MS04T17	lPGC	469	E50	genital ridge	130523ICSI-PGCAday50	female	GSE67259	5,167,957	**913,813**	306,932	49,705	3,442,011	455,496
GSM1643196	D50_MS04T14	lPGC	470	E50	genital ridge	130523ICSI-PGCAday50	female	GSE67259	8,528,249	**1,656,845**	524,818	77,031	5,716,794	552,761
GSM1643197	D50_MS04T15	lPGC	471	E50	genital ridge	130523ICSI-PGCAday50	female	GSE67259	5,334,342	**985,333**	496,368	31,348	3,319,044	502,249
GSM1643198	D50_MS04T16	lPGC	472	E50	genital ridge	130523ICSI-PGCAday50	female	GSE67259	8,113,939	**1,566,367**	485,739	55,837	5,306,171	699,825
GSM1643191	D43_MS04T06	lPGC	473	E43	genital ridge	130522ICSI-PGCday43	female	GSE67259	8,190,359	**3,428,491**	1,230,319	101,251	2,577,107	853,191
GSM1643194	D43_MS04T10	lPGC	474	E43	genital ridge	130522ICSI-PGCday43	female	GSE67259	19,071,477	**4,732,262**	1,885,120	213,117	10,580,768	1,660,210

**Table 2:** Summary of embryos and ESCs where single cells were picked from

**Embryo ID/Cell Line**	**sex**	**Embryonic Day/Passage number**	**cells picked from**	**cDNA constructed**	**good quality**	**SC3-seqed**
130206ICSI#1day6	male	E06	whole embryo	47	45	23
130227ICSI#1day6	female	E06	whole embryo	47	42	18
130306ICSI#7day6	female	E06	whole embryo	39	26	2
121128ICSI#1day7	male	E07	whole embryo	47	32	10
121205ICSI#1day7	male	E07	whole embryo	47	35	17
130319ICSI#1day7	male	E07	whole embryo	23	15	8
121107ICSI13_day8	male	E08	whole embryo	59	53	36
121212ICSI#11day8	male	E08	whole embryo	47	38	21
130220ICSI#11day8	male	E08	whole embryo	47	47	38
120725ICSIday9	male	E09	whole embryo	58	45	12
121212ICSI#2day9	female	E09	whole embryo	47	37	8
140812ICSId13A	male	E13	around embryonic disk	60	58	49
140401ICSIday14	male	E14	around embryonic disk	48	43	33
141014ICSIday14A	male	E14	around embryonic disk	60	58	30
140401ICSIday16	male	E16	around embryonic disk	48	46	36
140708ICSIday16	female	E16	posterior third of embryonic disk	96	94	8
140701ICSIday17	female	E17	around body stalk	96	92	3
140722ICSIday17A	male	E17	around embryonic disk	96	94	55
140401ICSIday20	male	E20	around body stalk	96	79	8
140708ICSId36#60	male	E36	genital ridge	48	13	6
130522ICSI-PGCday43	female	E43	genital ridge	58	55	7
130522ICSI-PGCday47	male	E47	genital ridge	60	34	1
130523ICSI-PGCAday50	female	E50	genital ridge	60	9	5
130522ICSI-PGCday51	female	E51	genital ridge	60	47	7
130522ICSI-PGCAday55	male	E55	genital ridge	30	17	1
130522ICSI-PGCBday55	male	E55	genital ridge	30	16	1
CMK9	female	p25	on feeder	24	9	3
CMK9	female	p30	on feeder	21	14	5
CMK6	male	p32	on feeder	46	32	10
CMK6	male	p33	on feeder	24	13	4
CMK6	male	p35	off feeder	24	3	9

**Table 3:** Primer list

**Gene**	**Primer Name**	**sense**	**anti-sense**
BMP4	mfBMP4-qPCR-s1/as1	ATTCCAGTATCCCCAAAGCCTG	GATCTCAGCGACACCCACATC
CDX2	mfCDX2-qPCR-s3/as3	CTTCCCAGACCAGGAAAGGC	GGCCTGGAGTCCAATAACCA
CER1	mfCER1-qPCR-s1/as1	GTAATTTGCTTGGTTGCCTCCT	CCAGTGATTGTTCCTCTTCCCT
COL6A1	mfCOL6A1-qPCR-s1/as1	CTTTTCCCACCAATCCTCACCT	CCTTGGTTTCCCAAAACGAGAC
DDX4	mmDDX4-qPCR-s1/as1	CATTCCTGGCTTCAGCGGTA	GGGTTGGGAGCTTGTGAAGA
DNMT3A	mfDNMT3A_qPCR-s3/as3	CTGCCAAAAAGGGGGCTAGA	CTGAAGACTCCGTACCCTGC
DNMT3L	mfDNMT3L-qPCR-s1/as1	CGGAAGAAGAATTGTCCCTGC	GGGAGAAAGCAGTTCTTCACCA
DPPA3	mfDPPA3-qPCR-s1/as1	AGGGATCTGTGTCATTTGAATGTAT	GGCTTCTAAGACACATGGGAATACT
FN1	mfFN1-qPCR-s1/as1	TGTGCAAGTCTCTCTAATTGTTGA	TTAAAGCAGAAAGTATAAGGCTGTC
FOXA1	mfFOXA1-qPCR-s1/as1	AGCTGGATTTGAAAATGTGGTCC	CCCGTCTGGCTATACTAACACC
FOXA2	mfFOXA2-qPCR-s1/as1	GAGGGTCACACTTGATACCCCAC	CAGGTGCTTGAAGAAGCAGGAG
FOXH1	mfFOXH1-qPCR-s1/as1	GAAACACCAGGCAGTAACCTTG	AGTGTAGGAGAGACAGAGGCAT
GAPDH	mfGAPDH-qPCR-s2/as2	AACAGGGTGGTGGACCTCAT	TTCCTCTTGTGCTCTCGCTG
GATA2	mfGATA2-qPCR-s1/as1	TTCTGTATGCGGTGATGGCCT	AGCGACTGCCCATCCATATT
GATA4	mfGATA4-qPCR-s1/as1	TGGCTATAGCAGAGAATACCTTTGAACCA	ACAGGTTTGTGGGTTAGGGAGGGT
GATA6	mfGATA6-qPCR-s1/as1	AGAAAGGCAATTTTCCTGAGAGA	ACATCCCATTTCTATATTTCCAATTGTT
IGF1	mfIGF1-qPCR-s1/as1	CCGCTGCTAAACACACCACAG	TAAGCTGCATGATATTTGAAAGGTTTTG
KRT18	mmKRT18-qPCR-s1/as1	CCAAGATCATGGCAGACATCCG	CTCTCCTCAATCTGCTGAGACC
LAMA5	mfLAMA5-qPCR-s1/as1	CGTCACCATGACTCGCTCTG	AGTGAGAGGAGTCACCTGCAA
MIXL1	mfMIXL1-qPCR-s1/as1	TTCAAAACACTCGAGGACTCCC	TGCTAAGGGTAACATTGGAGTGA
NANOG	mfNANOG-qPCR-s2/as2	TGTTCCGGTTTCCATTATGCC	TAGGCTCCAACCATACTCCA
NANOS3	mfNANOS3-qPCR-s1/as1	ATTTCCAGGAAGACCCACCCTA	GATTTCAGCTGTTGCCTCGC
PDGFRA	mfPDGFRA-qPCR-s1/as1	GCTGGCCTGAGAAACACAATTT	ACGTCCCTCTTCACAAAAATAGGA
POU5F1	mfPOU5F1-qPCR-s1/as1	GGGAGGAGCTAGGGAAAGAGAACCTA	CCCCCACCCGTTGTGTTCCCA
PPIA	mfPPIA-qPCR-s1/as1	ACAGGTCCTGGCATCTTGTC	CTCAGTCTTGGCAGTGCAGA
PRDM1	mfPRDM1-qPCR-s1/as1	TTCCCAACTACTCGTTTGTTCTTTG	CATGTAAGAGGCAGAAAAAGGAAGG
PRDM14	mfPRDM14-qPCR-s5/as5	TGCCCTGTTGTTTTAGGACTGT	AACCAGCAGTTAAGGAAAGGCT
SOX15	mfSOX15-qPCR-s1/as1	CCTAACCCACCTCTAACTCTGG	TGTAGTCCAACAGGAGAAAGGG
SOX17	mmSOX17-qPCR-s3/as3	TGCATTGTCAAAAACCCTATTTCCA	CACCCGGGACAACATTTCTTTG
SOX2	mfSOX2-qPCR-s1/as1	AAGGTTTTTCCCCCTTTATTTTCCG	GATTCTCGGCAGACTGATTCAAATA
SOX7	mfSOX7-qPCR-s1/as1	ATGATTGACGGGTGTGCAGTC	TGACAACTGTTTCAAAGTATGTGTCG
T	mfBRACHYURY-qPCR-s2/as2	TGCTGTCCCAAGTGGCTTAC	CTGGACCCTGGCAAACATCT
TCL1B	mfTCL1B-qPCR-s1/as1	ATAGATCCAGTGCTGAGCCAGT	TCGTGAGCTGAAGAACTGAACA
TFAP2C	mmTFAP2C-qPCR-s2/as2	TCGGAGATCAAGTCCTCTGG	CCTTTGAACACGGGGTTTAG
XIST	mfXIST-qPCR-s1/as1	TGTGAACTGATGTGAAATGCAGA	CCAACTCCCCAGTTTGTTTCAA
